# A Temporal Graph Model to Predict Chemical Transformations in Complex Dissolved Organic Matter

**DOI:** 10.1021/acs.est.3c00351

**Published:** 2023-05-09

**Authors:** Philipp Plamper, Oliver J. Lechtenfeld, Peter Herzsprung, Anika Groß

**Affiliations:** † Anhalt University of Applied Sciences, Department Computer Science and Languages, Lohmannstraße 23, Köthen 06366, Germany; ‡ Helmholtz Centre for Environmental Research − UFZ, Department of Analytical Chemistry, Research Group BioGeoOmics, Permoserstraße 15, Leipzig 04318, Germany; § ProVIS - Centre for Chemical Microscopy, Helmholtz Centre for Environmental Research - UFZ, Permoserstraße 15, Leipzig 04318, Germany; ∥ Helmholtz Centre for Environmental Research − UFZ, Department of Lake Research, Brückstraße 3a, Magdeburg 39114, Germany

**Keywords:** temporal graph, molecular network, compositional network, DOM, complex mixtures, photodegradation, photo-oxidation, link prediction, machine learning, community detection, unsupervised clustering

## Abstract

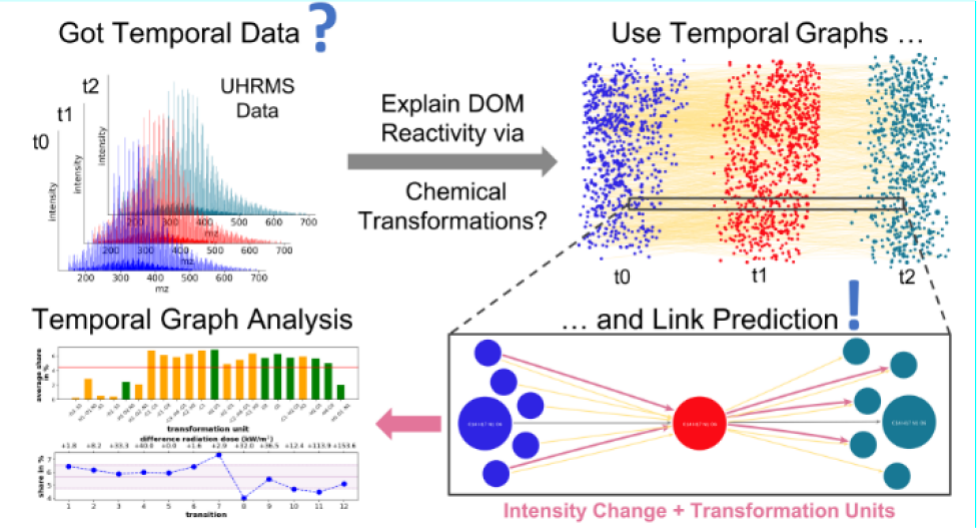

Dissolved organic matter (DOM) is a complex mixture of thousands of natural molecules that undergo constant transformation in the environment, such as sunlight induced photochemical reactions. Despite molecular level resolution from ultrahigh resolution mass spectrometry (UHRMS), trends of mass peak intensities are currently the only way to follow photochemically induced molecular changes in DOM. Many real-world relationships and temporal processes can be intuitively modeled using graph data structures (networks). Graphs enhance the potential and value of AI applications by adding context and interconnections allowing the uncovering of hidden or unknown relationships in data sets. We use a temporal graph model and link prediction to identify transformations of DOM molecules in a photo-oxidation experiment. Our link prediction algorithm simultaneously considers educt removal and product formation for molecules linked by predefined transformation units (oxidation, decarboxylation, etc.). The transformations are further weighted by the extent of intensity change and clustered on the graph structure to identify groups of similar reactivity. The temporal graph is capable of identifying relevant molecules subject to similar reactions and enabling to study their time course. Our approach overcomes previous data evaluation limitations for mechanistic studies of DOM and leverages the potential of temporal graphs to study DOM reactivity by UHRMS.

## Abstract 2

Temporal graphs are ideal to study the reactivity of complex mixtures like DOM in the environment and to reveal hitherto hidden processes.

## 1 Introduction

Dissolved organic matter (DOM) in surface waters is a highly dynamic component of the terrestrial carbon cycle and an important nutrient source for aquatic organisms.^1−4^ Due to its negative impact on color and odor and contribution to disinfection byproduct formation, it needs to be removed during drinking water production.^5,6^ Photochemical reactions of DOM play an important role in freshwater, as they modulate radiation (i.e., alter the available light), contribute to highly reactive species (reactive oxygen and hydroxyl radicals),^7,8^ and change the chemical composition of DOM during stratification periods in lakes or along the flow path of a stream/river.^9,10^ An increase in the number of sunny days, a widespread decline of spruce forests in temperate mountain ranges in middle Europe, as well as increased dissolved organic carbon (DOC) concentrations in many catchments have been reported.^11,12^ As a consequence, increased solar irradiation to previously shaded small streams and drinking water reservoirs and enhanced phototransformation of DOM are expected with currently unknown consequences for drinking water production from such waters.^13,14^

Although photochemical reactions of organic molecules are well-studied,^15^ the extreme chemical diversity of DOM and multiple reaction pathways make it challenging to identify and assess the importance of actual photochemical transformations in DOM.^16,17^ As a consequence, either only bulk parameters (DOC concentration) or photoreactivity indicators (reactive oxygen species, small molecular photoproducts) can be utilized to describe the extent and course of DOM photolysis.^18,19^ In order to gain insights about photochemical reactions in DOM (i.e., phototransformation of molecules via loss of small molecular fragments or oxidation) at the molecular level, ultrahigh resolution mass spectrometry, like Fourier-transform ion cyclotron resonance mass spectrometry (FT-ICR-MS), has been successfully applied,^20−23^ indicating in consistent changes in the molecular composition (i.e., high reactivity of electron rich systems like aromatic compounds and loss of CO_2_ as thermodynamically stable product). Similar approaches have been used for, e.g., halogenation, ozonation, or hydrogenation of organic matter, where few distinct molecular transformations can be identified based on specific mass and intensity differences.^24−27^ However, even in the most sophisticated experimental setups, only the net change in peak intensities can be evaluated (i.e., gain or loss of intensity of a compound represented by a molecular formula).^17,28^ This results in a lack of information about the pathway of transformation (i.e., complete mineralization or transformation into other molecules via elimination of small molecular fragments).

Due to the nature of DOM being a pool of molecules from many sources at different stages of degradation and reactivity, DOM can be considered as a complex network of compositional relationships.^29^ Compositional networks have been used for advanced visualization and statistical assessment of molecular compositions^30,31^ and molecular formula (MF) assignment and validation.^32^ Compositional networks are typically analyzed by counting the number of different chemical transformations within a sample (i.e., *intrasample*), quantifying their occurrence, and applying downstream statistics (e.g., via comparing the number of each transformation found within all samples). In metabolomics, metabolic networks are frequently used to analyze the metabolic state of organisms since the underlying biochemical reactions are well-known.^33,34^ However, this perception is likely an oversimplification of the highly dynamic and chemically diverse DOM pool. Major limitations are that the underlying processes are manifold (i.e., microbial production, adsorption), their rates are unknown, and that each process often only acts on a subset of the entire pool of molecules.^35,36^ As a consequence, compositional networks cannot be considered as static, time-independent networks but should be rather seen as temporal networks. Hence, compositional networks need to evolve into temporal networks (requiring time-resolved sampling as precondition), which has rarely been considered for DOM.

By definition, a network is a *graph* data structure and consists of *nodes* (objects), which are interconnected by various *edges* (relationships).^37^ A graph (network) is formally defined as G = (V,E), where V are the vertices, which we here refer to as nodes, and E are the edges connecting the nodes. Representing data as graphs is widely used to model real-world phenomena and capture knowledge from various domains.^38,39^ As such, graphs are not only the basis for specific visual representations of connected data but also represent the underlying data structure that forms the basis for a variety of graph algorithms such as path finding, centrality, or community detection. For DOM and metabolites, molecule nodes can be linked by directed edges to denote their chemical connectivity (via biochemical or other transformations) and thus to model chemical reactions. Applying ultrahigh resolution mass spectrometry techniques like FT-ICR-MS, MF can be derived from mass to charge ratios (*m*/*z*),^40^ allowing more detailed chemical description of the detected molecules. However, without directed edges, linking educts and products (e.g., via isotopic labeling), it is difficult to detect chemical transformation within complex mixtures like DOM. In particular, the lack of temporal properties in currently applied compositional network approaches for DOM hampers the determination of temporal trends in DOM composition.

A graph data structure combined with temporal properties would allow modeling and predicting directed edges *over time* and thus the evaluation of time-dependent changes in the DOM composition (e.g., via photolysis). Such a *temporal graph* can be particularly useful in identifying new links between the entities (i.e., molecules) in the considered data.^41−47^ Using graph analytics tools such as link prediction, graph-based clustering, and visual analysis, it is possible to identify hidden patterns in the data, such as the relation between change in intensity of a meaningful subset of MF and transformations likely responsible for this change. Better assessment of the actual occurring chemical transformations in complex DOM would help to better evaluate natural processes as well as anthropogenic impacts constantly altering the molecular composition and chemical properties of DOM.

We present a novel approach based on a temporal graph model to predict chemical transformations of DOM using molecular data obtained by FT-ICR-MS. The temporal graph approach shall enable us (1) to better represent the highly dynamic nature of the DOM and (2) to gain new insights on underlying chemical reactions and their temporal dynamics based on graph analytics. To achieve this goal, we developed a dedicated graph model and a link prediction algorithm that identifies directed temporal edges between molecules over consecutive time points by considering the changes in the normalized intensities of educts and products. The graph model and algorithm were implemented in a graph database management system, and the resulting graph was further analyzed visually and statistically. For the model development and proof-of-concept, we used a previously published data set of a high-resolution photodegradation experiment representing a simplified experimental system.^20^ The model enabled us to derive new information about photochemical reactivity of DOM based on an evaluation of the predicted transformations, and the results are cross-validated based on the previous data evaluation.

## 2 Materials and Methods

To develop and validate the new approach to study DOM reactivity based on a temporal graph, we used data from a photolysis experiment previously published by Wilske et al. (2020).^20^ Briefly, water was collected from a drinking water reservoir inflow. The water was filtered and exposed to natural sunlight for 6 days and analyzed with direct infusion negative electrospray ionization FT-ICR-MS at 13 time points during the experiment. This data set is a particularly useful test case for the temporal graph approach as it (i) utilizes natural water and natural sunlight, (ii) mostly excludes other DOM transformation (like microbial degradation or sorption), (iii) has a sufficient temporal resolution to resolve temporal dynamics within the system (e.g., rapid versus slow degradation of some compounds or detection of new (transient) compounds), and (iv) has already been evaluated in a robust statistical manner, allowing for cross-validation of results.^20^ Experimental and data processing details are described in the Supporting Information (SI Text Experimental Setup and Data Preparation). To construct the graph of chemical transformations in the photolysis experiment, we used 22 photochemical reactions compiled and described by Hu and co-workers (SI Table 1).^17,28,48−52^ Details about the transformations and related assumptions can be found in the SI Description Photochemical Transformations of DOM and SI Table 1. Importantly, for this study, we differentiate between *photo elimination* (product with lower molecular weight, MW) and *photo addition* (product with higher MW). At first, all selected transformations are considered *potential transformations*. Using the temporal information and peak intensity changes, we further specify a subset of transformations as*predicted transformation*s, as outlined below.

### 2.1 Temporal Graph Model

We developed a temporal graph model to accurately describe the multidimensional molecular and temporal data from the photodegradation experiment. Based on the model, we created a large graph of molecules which allows for intelligent graph-based analytics on the data such as the prediction of transformations within DOM or the identification of molecule clusters.

Our temporal graph is a so-called labeled property graph,^53^ a commonly used model in graph databases.^54^ Nodes are labeled with a type (in our case “Molecule”) and further enriched with properties such as MF and peak intensity. The edges in the property graph are described by a label and can accommodate more properties, such as weighting factors. Our graph model further belongs to the group of the snapshot-based temporal graphs,^42,46,55,56^ where one graph exists for a discrete point in time, reflecting here the discrete sampling times from which DOM compositions were analyzed. These single graphs or snapshots are interconnected with other snapshots via temporal edges to build the temporal graph.

**Figure 1:**
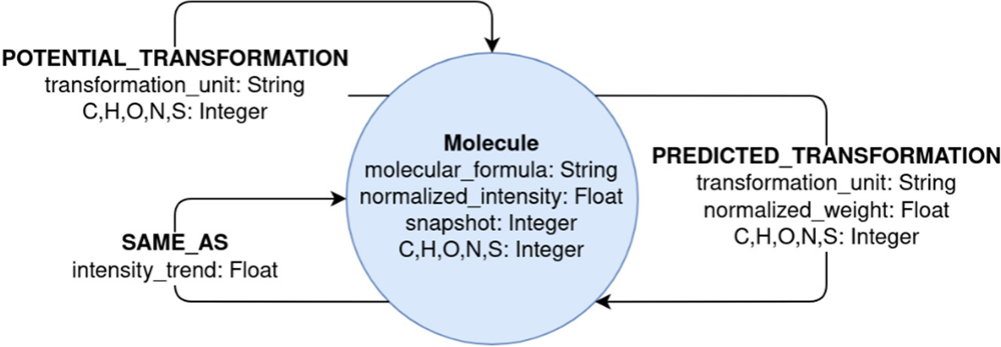
Temporal graph model with nodes, edges, and their properties. The model contains static (e.g., molecular formula (MF), transformation unit) and temporal (e.g., snapshot, normalized intensity) properties. The start and end of any edge is always a node of type “Molecule”. Since the same MF can appear in multiple snapshots of the graph, a node is uniquely identified by the combination of the MF and the snapshot.

The schema of our temporal graph model for complex chemical networks, such as photochemical reactions of DOM is shown in Figure 1. We considered only one object type −“Molecule” − with added properties “molecular_formula” and the respective number of atoms for each element as well as the temporal properties “snapshot” and “normalized_intensity”.

The model interconnects molecules by three different types of temporal edges. Same molecules (i.e., the same MF) appear as often in the temporal graph as they were analyzed during the photodegradation experiment. They can be distinguished by their snapshot property, which reflects the experimental time point and ranges from 1 to 13. The snapshot property can also be used to distinguish and order the single snapshots in the temporal graph. First, to connect molecules with the same MF in consecutive snapshots, we define the “SAME_AS” edges. These edges contain as property the ratio of the normalized intensities of the connected molecules (intensity of later node divided by intensity of earlier node), which is termed “intensity_trend”. Second, we define edges that describe the potential chemical transformations. These edges always start from a molecule in one snapshot and end at another molecule in a consecutive snapshot and are denoted as “POTENTIAL_TRANSFORMATION” edges. “POTENTIAL_TRANSFORMATION” edges are described by a transformation unit property reflecting the underlying molecular formula differences (SI Table 1). The edges are calculated in advance by a developed algorithm (SI Algorithm 1). Depending on the mass difference the transformation either belongs to the photo addition or photo elimination group. In this step, we omit isolated nodes without any “POTENTIAL_TRANSFORMATION” edges (here: 0.3% of all nodes). Lastly, we define edges that describe predicted likely occurring chemical transformations. These edges are a subset of the “POTENTIAL_TRANSFORMATION” edges and indicate only those chemical transformations that likely occurred during the photodegradation experiment based on the normalized intensity differences. We denote them as “PREDICTED_TRANSFORMATION” edges. The calculation of the “PREDICTED_TRANSFORMATION” edges follow further specific rules and considerations and is described in more detail in Section 2.2.

**Figure 2:**
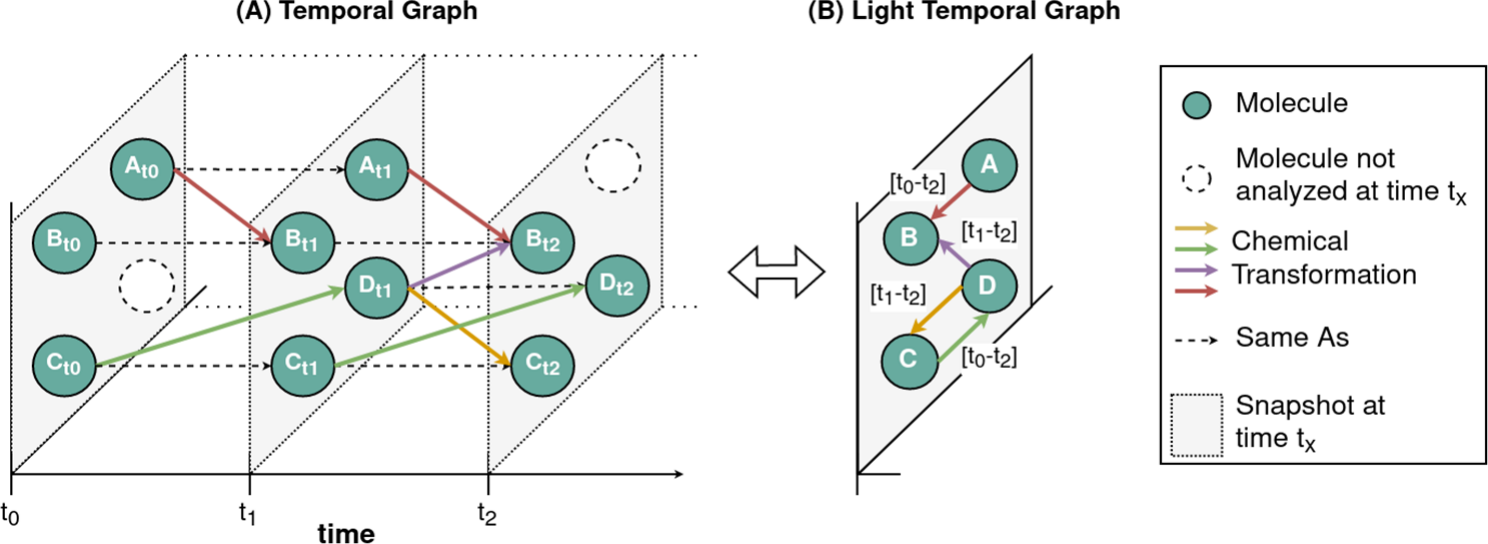
(A) Example of the temporal graph with 3 consecutive snapshots (*t*_0_, *t*_1_, *t*_2_) and four different molecules (A–D). Every snapshot differs with respect to the molecular composition and some molecules are only present in some snapshots (e.g., D_t1_, D_t2_), while others occur in multiple snapshots (e.g., B_t0_, B_t1_, B_t2_). The edges are directed and start on a node in one snapshot and end on a node in the subsequent snapshot. Edges with the same color describe the same chemical transformation (e.g., from C to D), which can occur only once or multiple times. Note that in the temporal graph, nodes have no edges to other nodes in the same snapshot and nodes without any edge are omitted. (B) Conversion of the original temporal graph to the light temporal graph that stores the same information. The light graph allows utilization of smart graph algorithms like community detection not possible with the original graph.

To illustrate the use of the temporal graph model, Figure 2A shows the interrelation between three exemplary snapshots, each with a molecular composition and related chemical transformations. Note that in our model no edge exists between two molecules within the same snapshot and that while the snapshots are numbered with integer values, they may represent nonequidistant time points (here: duration of experiment upon sampling) or reaction progress (here: accumulated radiation in kW/m^2^).

The temporal graph model is suitable for graph exploration, such as interactive queries to search for specific patterns of reactions or traversing the molecule transformations over time. However, many graph algorithms (such as community detection or centrality algorithms) have originally been developed for static graphs and there is a need for advanced algorithms on temporal graphs.^57−61^ To apply graph algorithms suitable for homogeneous graphs, our temporal graph can be converted to its *light* representation (Figure 2B). The *light temporal graph* uniquely represents every molecule and combines aggregated temporal transitions into one edge type. Still, the light version of the temporal graph stores the same information, and a conversion in both directions is possible without information loss. To meet the requirements for static graph algorithms, the edge types and their properties (e.g., intensity trend, transformation unit, predicted transformation) as well as temporal properties of the nodes (e.g., normalized intensity, snapshot) are stored as properties in the new “CHEMICAL_TRANSFORMATION” edges of the *light* temporal graph (SI Figure 2). Those edge properties are useful as weights adopted by graph algorithms.

Based on our temporal graph model, we implemented the graph in the open-source and native graph database Neo4j and Python (for details, see SI Text Implementation of the Graph Model, SI Description 1, and SI Algorithm 1). The open source project is called *tegrom* (**Te**mporal **Gr**aph Model on D**OM**) and is publicly available on GitHub (https://github.com/PhPlam/tegrom).

### 2.2 Predicting Chemical Transformations

**Figure 3:**
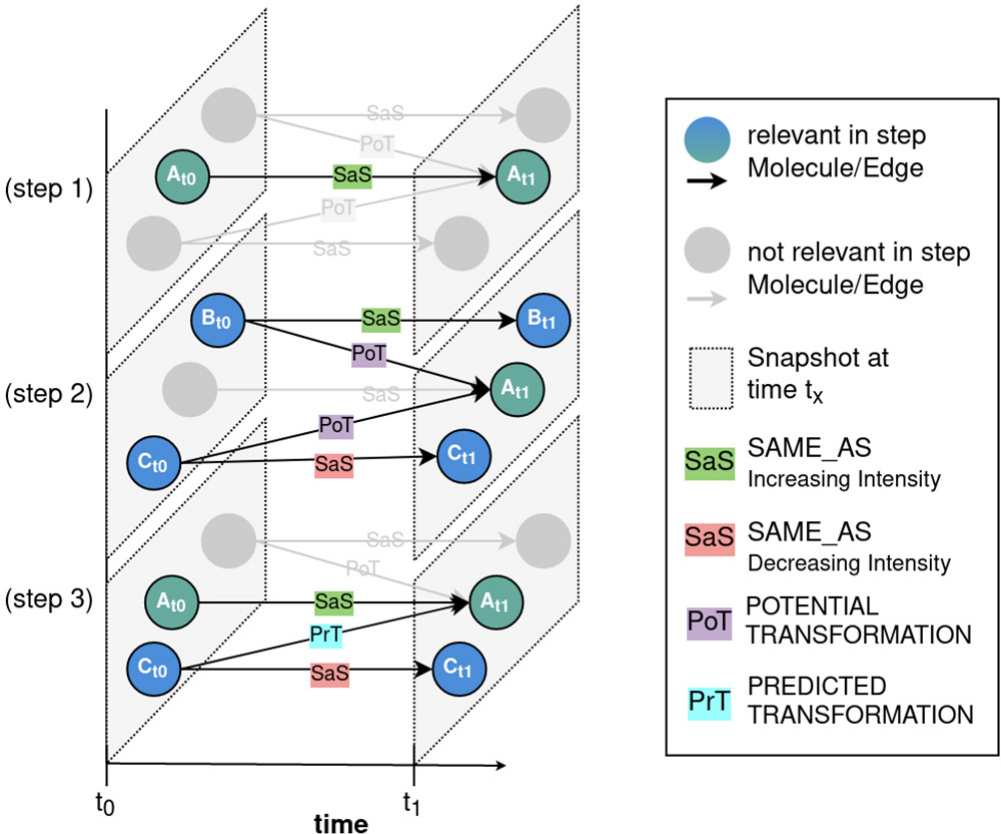
Iterative steps of the “Transformation Prediction Algorithm” to predict the likely occurring chemical transformations: (step 1) Get incoming “SAME_AS” edges. Continue if the intensity trend increases. (step 2) Get the incoming “POTENTIAL_TRANSFORMATION” edges and their intensity trends. (step 3) Remove the nodes with an increasing intensity trend and add the remaining “POTENTIAL_TRANSFORMATION” edges as “PREDICTED_TRANSFORMATION” edges in the graph. See SI Description 2 and SI Description 3 for a detailed description of the considerations and steps.

So far, the temporal graph contains all the necessary information describing the molecular and temporal data from DOM analyses of the photodegradation experiment. Knowing about the direction of chemical reactions and assuming that a consumption (production) of an educt (product) will be reflected by a decrease (increase) in its normalized peak intensity it is possible to retrieve those chemical transformations that are likely responsible for the temporal dynamics within DOM. We therefore developed the “Transformation Prediction Algorithm” (TPA) to predict likely occurring chemical transformations based on already established potential chemical transformations and intensity trends using three considerations (SI Description 2, SI Figure 3). In short, the “PREDICTED_TRANSFORMATION” edges are a subset of all “POTENTIAL_TRANSFORMATION” edges and start always on a node from snapshot t_*x*_ with a decreasing intensity trend to snapshot t_*x*+1_ at the “SAME_AS” edges and end on a node at snapshot t_*x*+1_ with an increasing intensity trend at the respective “SAME_AS” edge from snapshot t_*x*_ to t_*x*+1_. Figure 3 illustrates the main steps of the TPA. It is important to note that the node in snapshot t_*x*_ and the node in snapshot t_*x*+1_ can only be connected by a “PREDICTED_TRANSFORMATION” edge, if both nodes display opposite intensity trends which are larger than the considered measurement error margin (here 5%, cf. SI Text Experimental Setup and Data Preparation). In other words, the normalized intensity difference of the educt and product molecules must be larger than 5%. In order to cover all nodes in all snapshots, the considerations in the TPA are implemented as an iterative algorithm (SI Description 3, SI Algorithm 2), starting with the assessment of the intensity trends at the “SAME_AS” edges.

We validated the general applicability of the approach and model with an independent photochemical irradiation experiment of a distinct compound with known phototransformations (cf. SI Text Model Validation with Carbamazepine).

One metric derived from the TPA are weighting factors for the “PREDICTED_TRANSFORMATION” edges. The weight was calculated using the intensity trends at the “SAME_AS” edges and the normalized intensity of the nodes (SI Text Calculation of the Weights at the “PREDICTED_TRANSFORMATION” Edges). Molecules are now interconnected with predicted transformations over time. To analyze the structure of the DOM network, it is useful to identify clusters of molecules, e.g., to get insights on potentially hidden structures in the graph. As an initial approach, we used the community detection algorithm *Label Propagation*(62) (LPA) provided by Neo4j^63^ on our light temporal graph to cluster the graph based on edges and their connectivity in the graph. The algorithm does not require predefined information about potential clusters. It assigns labels (imagined as colors) to the nodes in the graph and propagates them through the network structure. Within an iteration, every node updates its current label to the label of the connected nodes with the highest edge weight. The algorithm terminates when the states of the groups no longer changes. The resulting communities can provide information about edges inside and outside the groups or about dense and sparse regions in the graph. In our temporal graph, the algorithm uses the number of predicted chemical transformations between molecules as the edge weight such that molecules with a high edge weight are more likely to be in the same group.

## 3 Results and Discussion

To test the graph models and the TPA and demonstrate their suitability for DOM transformation evaluation, we used the FT-ICR-MS data from the photochemical experiment of Wilske et al. (2020)^20^ and the results of the predicted likely occurring chemical transformations.

### 3.1 Temporal Graph Structure

**Figure 4:**
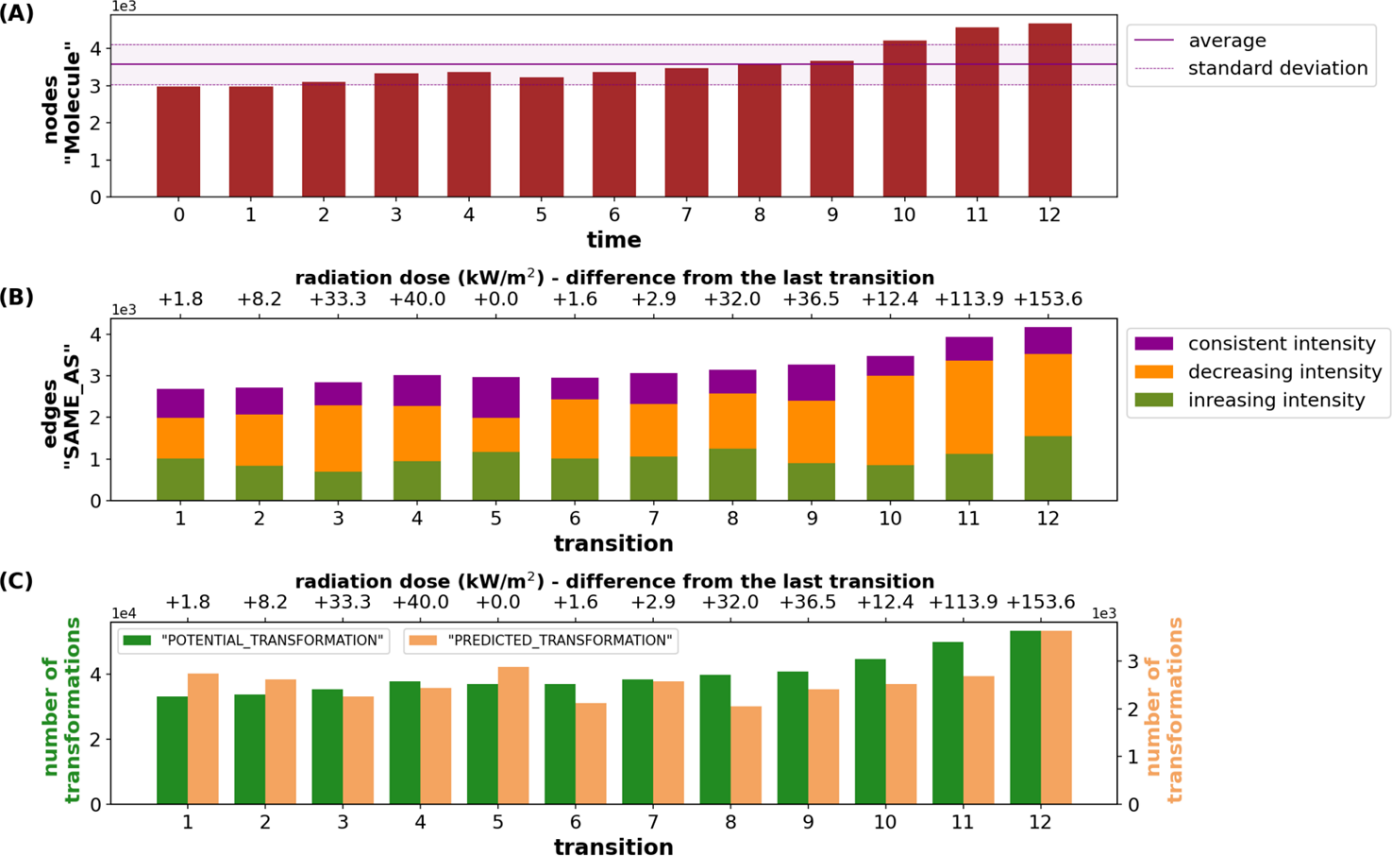
Key metrics of the temporal graph. (A) The number of nodes with the label “Molecule” per snapshot (*n* = 13). (B) Distribution of intensity trends (divided into consistent, decreasing and increasing) at the “SAME_AS” edges between two consecutive snapshots (transitions). (C) The number of *potential* and *predicted transformation* edges describes the chemical transformation per transition. Note that for *n* snapshots only *n* – 1 transitions can be calculated.

The developed temporal graph model transforms the table structure of the experimental data into a feature-rich complex graph structure (SI Figure 4). Examples of subgraphs with *predicted transformations* for a selected MF across all transitions are displayed in SI Figure 5. The number of nodes in the temporal graph ranged between 3000 and 4700 and increased toward later snapshots (Figure 4A). This reflects an increase in the number of detected MF at the end of the time series, also observed in other photodegradation experiments using mild irradiation, where new molecules appear as a result of cleavages.^22,50^ The number of “POTENTIAL_TRANSFORMATION” edges ranged between about 33000 and 53000 and also increased with the number of nodes (Figure 4C). This is expected for complex organic matter and is also demonstrated by compositional networks.^31,32,64^

The number of “SAME_AS” edges also increased from about 2700 to about 4200 (Figure 4B), reflecting that the majority of molecules were detected in more than one sample (SI Figure 1). Note that since not all MF are detected in all samples, the number of “Molecule” nodes was larger than the number of “SAME_AS” edges for every snapshot. With an average of about 1500, most nodes show a decreasing intensity trend, 1000 an increasing and 700 a consistent intensity trend. The number of “PREDICTED_TRANSFORMATION” edges varied between 2000 and 3600 (Figure 4B, C), resulting in an overall mean of 0.74 *predicted transformations* per molecule and transition. The distribution of the outgoing “PREDICTED_TRANSFORMATION” edges per node (SI Figure 6) shows that many nodes have very few outgoing edges, while only a few nodes have many outgoing edges. Networks that follow this power-law distribution are known as scale-free and are typical for this type of network.^32,33,65^ At the level of individual MFs, the distribution of “PREDICTED_TRANSFORMATION” edges can also largely vary depending on the MF and transition (SI Figure 5).

In contrast to the *potential transformations*, the *predicted transformations* are based on intensity changes of the MF between two snapshots, which, in turn, may reflect the dose of incident radiation. Although statistically not significant, the number of *predicted transformations* increased with the irradiation dose between snapshots (Figure 4C). Consequently, when considering the intensity trends at the “SAME_AS” edges between consecutive snapshots, the proportion of decreasing, increasing, and unchanged MF intensities also varied, with more decreasing edges related to a higher irradiation dose between snapshots (Figure 4B). Expectedly, during the first night (transition 5, ΔkW/m^2^ = 0), a larger proportion of “SAME_AS” edges showed a consistent intensity, whereas toward the end of the experiment, more molecules showed a decreasing intensity, likely reflecting the much larger irradiation doses for the transitions 11 and 12 (Figure 4B).

### 3.2 DOM Transformation Analyses with the Temporal Graph

**Figure 5:**
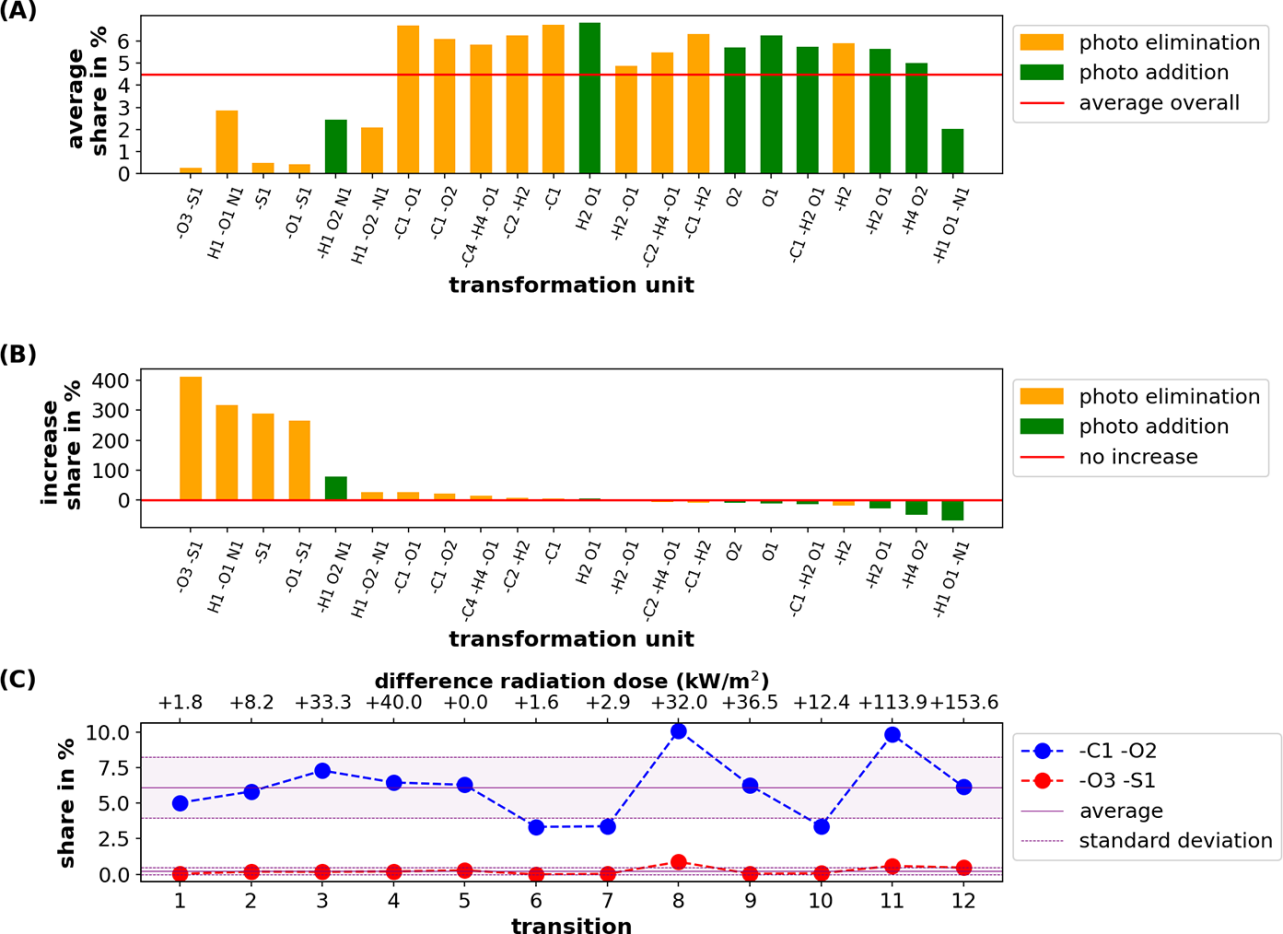
(A) Average proportion of transformation units according to all *predicted transformations* and (B) their relative change (% increase or decrease) in proportion from the first to the last snapshot during the photodegradation experiment. Transformation units are grouped into *photo addition* (green) and *photo elimination* (orange) according to the change in molecular weight. The red line in (A) indicates the grand mean for all transformation units and transitions. (C) Trend of the share of transformation units “-CO2” and “-SO3”. Both transformation units belong to the *photo elimination*s. “-SO3” has a much lower average share than “-CO2” but increases more across the snapshots.

One key quantitative measure of the temporal graph is the proportion (share) to which each *predicted transformation* contributes to the overall compositional change (Figure 5A). This allows detailed temporal analyses for each transformation unit, e.g., observing trends within and across *predicted transformations* (Figure 5B,C, SI Figure 7). In addition, by using the degree of relative intensity change between educts and products (and accounting for all of the transformation from/to them) as weighting factors, relative importance metrics for each transformation can be derived (SI Figure 8).

*Photo elimination* transformations like decarboxylation (“-CO2”), which is also driving DOC loss, or dehydration (“-H2O”) are mechanistically important reactions in photolysis experiments.^20,23^ Elimination reactions with only C, H, and O are frequent (Figure 5A) but show little variability overall (Figure 5B). Products of these eliminations may be CHO MF or (other than oxygen-)heteroatom-containing MF (e.g., CHNO, CHOS), allowing for downstream reactions with alike transformations units. In addition, it is more likely that a heteroatom-containing product like CHNO or CHOS was not initially present, due to the overall lower number of CHNO and CHOS MF. This is in contrast to heteroatom-containing transformation units (e.g., “-SO3”) which have a lower share across all transitions (Figure 5A), reflecting the lower number of heteroatom-containing molecules in all samples (SI Figure 9), but an importance (i.e., contribution to MF intensity change) well above average (SI Figure 8). The product of transformations like “-SO3” is often an MF without heteroatom, limiting the number of such transformations to the relatively low number of heteroatoms in DOM-MF. Together, this can also explain why the number of CHNO and CHOS MF increases more than CHO MF and their fraction even increases despite some specific eliminations with heteroatoms (SI Figure 9).

*Photo addition* transformations, although fewer, also play an important role in photolysis of DOM, as they represent widespread photo-oxidation reactions responsible for photo bleaching.^66^ Despite their importance, *photo addition* transformations more often decrease, whereas *photo elimination* transformations mostly increase over time (Figure 5B). This can be explained with the fact that oxidation of alcohols and aldehydes may only proceed to a carboxylic acid (“-H2+O” or “+O”). This may be followed by cleavage of the acid as “-CO2”, which, however, is a *photo elimination*. Similar considerations apply to the oxidation of double bonds. An exception is “+NO2-H″, representing a photoinduced nitration (e.g., of aromatic moieties), which increases during the experiment (Figure 5B) and has a high importance based on its impact on intensity change (SI Figure 8). Photolytic incorporation of inorganic nitrogen into DOM has been discussed previously.^50^

Considering the broader classification into *photo addition* (increasing MW) and *photo elimination* (decreasing MW) transformations, the overall share of *photo elimination* transformations is mostly higher than the share of *photo addition* across all transitions (SI Figure 10). This is in agreement with the fact that the intensity weighted average MW decreased over the course of the experiment and the findings from Wilske et al. (2020) showing that larger molecules tend to be preferentially degraded.^20^

A detailed analysis of the trends of individual transformations revealed transformation patterns, which are related to the irradiation dose for each transition. The number of decarboxylation transitions (“-CO2”) was below the mean for low irradiation doses (transitions 1, 2, 6, 7, 10) and showed distinct maxima with high irradiation doses (transitions 3, 4, 8, and 11; Figure 5C). The number decreased again toward the end (transition 12), possibly indicating an exhaustion of the sample with respect to potential CO_2_ losses as also confirmed by a decreasing DOC concentration (approximately 30% loss of DOC; cf. Table S2 in ref (20).).^20^ In contrast, the photo-oxidation transformations (“+O”, + “O2”, and “-H2+O”) tended to have a larger-than-average share for the low radiation doses (during sunrise at days 1, 2, and 3) and below average for the higher irradiation doses during midday and the final sampling points (SI Figure 7). This is consistent with the pattern from the intensity weighted average molecular descriptors, showing an initial increase in weighted average O/C ratio (expected for “oxidation”), followed by an increase in H/C and decrease in O/C (expected for a loss of CO_2_) (SI Figure 11).

Overall, the analysis of the temporal graph based on selected transformation units and intensity changes of the underlying MF matches well with the perception from the aggregated data of the individual samples. However, clear benefits of the graph model are an immediate evaluation of the importance of different transformations for the observed changes in peak intensities (vs simple change in MF intensities with unresolved reaction pathways) as well as easy access to their trends and time course achievable with interactive queries on the graph database. The simultaneous consideration of all intensities across two snapshots is another advantage over simple linear regression models as it avoids type-II errors and increases the robustness of the evaluation.

### 3.3 Temporal Graph Clustering

MF can be connected via similar sets of “PREDICTED_TRANSFORMATION” edges. To identify clusters of MF, we applied the *Label Propagation Algorithm* (LPA) on the light temporal graph. Here, clusters are characterized by sets of transformation units, which connect the educt and product MFs. In contrast to common clustering approaches for DOM, here we use the predicted chemical transformations as well as their absolute counts (i.e., occurrence) across all snapshots as a basis for the clustering, not the similarity of normalized intensity values of the MFs itself.

The top four clusters from the LPA comprise 60% of all unique MF in the data set (SI Figure 12). The first cluster (Cluster 1) with most MF (*n* = 1945, 35%) also contained the majority of unique CHNO MF in the data set (*n* = 1060, 62%). This cluster was characterized by a broad distribution of MF in the chemical (H/C vs O/C) space. Notably, this cluster also represented most transformation units involving N among all clusters with a relative share of 14% of all transformations in this cluster. A subset of the CHNO MF in cluster 1 was previously identified as photoproducts (SI Figure 13). Considering the main transformations in cluster 1 involving nitrogen (“+O-NH”, deamination, and “+NO2-H”, nitration), which also have a high importance, the MF identified as educts are distinctly different with respect to their H/C and O/C ratios, reflecting their presumed reactivity (SI Figure 8, SI Figure 14).

The majority of MF in Clusters 2–4 were CHO MF (SI Table 2). Notably, Cluster 2 (*n* = 560), although being characterized by more saturated (higher mean H/C) and less oxygenated (lower mean O/C) MF (SI Figure 12A, SI Table 3), had a higher share of CO and CO_2_ loss as compared to Cluster 1. Cluster 3 (*n* = 436) and 4 (*n* = 393) which comprised MF with distinctly higher mass (SI Figure 12B), were characterized by a larger share of photo-oxidation reactions (+O, -H_2_+O, Σ14%) as compared to Clusters 1 and 2 (Σ11%), likely corresponding to a higher likelihood of alcohols and aldehydes in larger molecules. Cluster 4 had a lower nominal oxidation state of carbon and lower aromaticity index than cluster 3, which is consistent with the fewer detected “-CO2” transformations (4.6%) compared to cluster 3 (6.8%; SI Table 4).

Out of all 1478 MF shared with the data from Wilske et al. (2020), 1458 MF (97%) were represented by the first four clusters of the LPA (SI Figure 15). The largest overlap was found between cluster 1 and the “-0.001” group (335 MF, 23%) and between cluster 2 and the nonsignificant MF (304 MF, 21%; SI Figure 13, cf. Figure 6 in Wilske et al. (2020)). This indicates that the LPA indeed assigns groups based on the predicted transformations, rather than overall trends in MF intensity (as e.g., derived from rank correlation analysis in Wilske et al. (2020)). This is further supported by the fact that the mean intensity trends of all “PREDICTED_TRANSFORMATION” edges are always close to one (SI Table 2), indicating that each cluster contains both educts (which decrease in intensity for any given transformation) and products (which correspondingly increase in intensity), linked by the “PREDICTED_TRANSFORMATION” edges. Some MF which were classified as nonsignificant in the previous study could now be attributed to a cluster (i.e., cluster 1) indicating a similar reactivity of such MF as compared to those with highly significant trends of their overall intensity (SI Figure 13).

Overall, the use of community detection algorithms like the LPA is highly beneficial for the temporal graph analysis, as it was able to detect clusters of MF sharing common transformations. The detected clusters of MF (considered dense) are distinguishable on the basis of their elemental composition and chemical properties and have clear boundaries with other clusters. The boundaries of the detected clusters consist of regions that are less connected (considered as sparse). Implementation of further community detection algorithms and analysis of the clusters could help to refine the mechanistic understanding of the underlying reactivity during photolysis of DOM.

### 4 Benefits, Limitations, and Further Applications for Temporal Graphs

The developed snapshot-based temporal graph model transforms the tabulated data structure of ultrahigh resolution mass spectrometry measurements of DOM into a feature-rich complex and highly connected - yet intuitive, graph structure. The nodes and edges store not only static but also time-dependent properties inherent in chemical transformations. The implementation of the temporal graph in a graph database such as Neo4j enables fast and efficient queries and traversals of the temporal data. This aids in the visualization and understanding of the complex interactions and transformation processes in experimental setups with the DOM.

Starting with a set of knowledge-based, expected reactions (i.e., potential transformations), our link prediction algorithm calculates new edges between nodes representing the likely occurring predicted transformations. Our approach extends previous compositional network analysis approaches (which consider edges within a sample) by including multiple measurements of the same sample at different experimental times. Specifically, we make use of changes in peak intensity, which have previously been shown to systematically vary according to the applied irradiation dose.^20^ It is now possible to distinguish more likely and less likely transformations on the basis of the direction of edges and intensity variation between educts and products. However, the assumption that a change in peak intensity always represents a change in compound abundance may be challenged based on the sample matrix and signal-to-noise ratios of underlying mass peaks,^67,68^ but has been shown to produce robust results in simplified experimental setups.^20,35^ Experimental or measurement variability also needs to be taken into account e.g. via sample replication and sensible adjustment of error margins used as input of our TPA. Furthermore, even with higher temporal resolution of the acquired data, only net reactivity between two consecutive snapshots can be considered by the graph model, hampering further mechanistic and kinetic assessment of transformations in complex DOM samples. However, the predicted transformations and derived importance metrics (frequency, weights) represent predominant reactions that largely change the molecular composition of the sample and thus can guide further targeted assessment of the individual DOM molecule reactivity.

By modeling DOM transformations as temporal graphs, machine learning graph algorithms (e.g., *Label Propagation* community detection) can be used to derive clusters of molecules with similar reactivity enriched with temporal properties as weights. Using direct infusion MS data (i.e., no isomer resolution), the study of DOM transformation is limited to the molecular formula level, knowing that DOM reactivity may indeed vary based on isomers. Other novel approaches, e.g., involving isotopic labeling or tandem mass spectrometry, are used to find structural commonalities and dissimilarities among different samples.^69−71^ These approaches are ideal to further constrain the (so far only presumed) molecular connectivity within a sample and add further chemical knowledge to the common compositional network approaches. As such, they are also suited to extend our temporal graph approach to predict transformations by adding further constraints which transformation may happen or not, depending on the functional groups present.

The temporal graph model presented in this study is not limited to photochemical reactions and can readily be extended to other processes acting on DOM where complex reactions impede detailed mechanistic studies but knowledge on transformation pathways already exists (e.g., ozonation, disinfection of drinking water, microbial degradation, or redox reactions) and no isotopic tracer can be employed.^21,24,72^ In combination with dedicated high-resolution experimental setups and environmental monitoring,^35^ the impact of climate change related shifts in catchment biogeochemistry and implications for (drinking) water quality can be better addressed, if the dynamic nature of these systems is appropriately reflected also in the data evaluation, e.g., via temporal graphs.

Moving forward, temporal graphs can leverage nontargeted mass spectrometry of complex mixture to a new level of information extraction. This is urgently needed to increase the value of nontargeted mass spectrometry to contribute to solving complex environmental problems where multiple processes occur simultaneously.^73^

## Supporting Information Available

Details about the experimental setup, data processing, selection of transformation units, algorithm and rules for the “Transformation Prediction Algorithm”, a summary of the data set, the predicted transformations and clusters, visualizations of the model and resulting graph, and a detailed comparison with the results from the previous study by Wilske et al. (2020).^20^ (PDF)
