# Supplementary material for: A Temporal Graph Model to Predict Chemical Transformations in Complex Dissolved Organic Matter
Source: Environ Sci Technol. 2023 May 9;57(46):18116–26. doi: 10.1021/acs.est.3c00351 (PMC10666529; doi:10.1021/acs.est.3c00351)
Supplement: Supplementary file 1 — es3c00351_si_001.pdf [file es3c00351_si_001.pdf]

Supporting Information for

A temporal graph model to predict chemical transformations in complex dissolved organic matter

Philipp Plamper[1], Oliver J. Lechtenfeld[2,3,], Peter Herzsprung[4], Anika Groß[1,*]*

[1] Anhalt University of Applied Sciences, Department Computer Science and Languages, Lohmannstraße 23, 06366 Köthen, Germany

[2] Helmholtz Centre for Environmental Research – UFZ, Department of Analytical Chemistry, Research Group BioGeoOmics, Permoserstraße 15, 04318 Leipzig, Germany

[2] ProVIS - Centre for Chemical Microscopy, Helmholtz Centre for Environmental Research - UFZ, Permoserstraße 15, 04318 Leipzig, Germany.

[4] Helmholtz Centre for Environmental Research – UFZ, Department of Lake Research, Brückstraße 3a, 39114 Magdeburg, Germany

Number of pages: 27

Number of Texts: 5

Number of Descriptions: 3

Number of algorithms: 2

Number of tables: 4

Number of figures: 19

SI Text

Experimental Setup and Data Preparation

Experimental details are described in Wilske et al. (2020).[1] Briefly, DOM collected from a drinking water reservoir inflow was filtered (0.2 µm GF/F, Whatman) and subjected to natural sunlight in quartz flasks for a total of six days, resulting in a total accumulated radiation dose of 436 kW/m² after 145 h (including dark periods). Samples were collected at irregular time intervals according to the daily radiation profiles and expected degree of DOM transformations (i.e., 0, 2, 4, and 8 h after sunrise during the first two days and only once per day thereafter). Samples were then filtered (0.2 µm GF/F, Whatman), extracted (via solid phase extraction, PPL, 50 mg, Agilent, Waldbronn, Germany), and analyzed with direct infusion negative electrospray ionization FT-ICR-MS according to established methods as described in Wilske et al. (2020).[1]

For this study we used the original data (after molecular formula assignment, 150-750 Da, $C_{1-60}H_{1-122}O_{0-40}N_{0-2}S_{0-1}$), sum normalized peak intensities and results from the spearman rank correlation of mass peak intensities and accumulated radiation dose, dividing MF into distinct groups according to the monotonic time course during the experiment (i.e., a photo product being a MF that consistently increased with ongoing radiation).

Deviating from the published data (using the shared molecular formulas in all samples), here we used all MF present in averages from experimental replicates (using the arithmetic mean of the peak intensities) after blank subtraction (resulting in 5521 unique MF) in order to account for potential transient compounds (e.g., MF not detected at the end). Also, the graph model does not require MF to be present in all samples (i.e., time points, SI Figure 1), nor does it assume monotonic behavior of peak intensities. Accounting for the analytical uncertainty of measured peak intensities in FT-ICR MS, we use a minimum of 5% normalized intensity change as significance threshold (cf. SI Algorithm 2), resulting in intensity trend values (ratio of peak intensities) considered as consistent between 0.975 and 1.025, as increasing for >1.025, and as decreasing for <0.975. Note that in this case we use a relatively narrow error range, since individual triplicate measurements were already averaged.

Photochemical Transformations of DOM

To construct the graph of chemical transformations in the photolysis experiment, we compiled common photochemical reactions observed for organic molecules, which have also been discussed to be relevant for DOM (SI Table 1).[2] These include reactions related to common DOM photoproducts (e.g. CO, CO_2), as well as reactions of functionals groups with other heteroatoms than O, like sulfonic acids (desulfonylation) or amines (deamination), known to release sulfate and ammonia, respectively.[3–8]

Importantly, the used transformations do not strictly represent singular molecular pathways (i.e., mechanistically resolved), but rather net reactions which may require multiple reaction steps occurring at time scales well below the temporal resolution of the experiment. Hence, we use the term *transformation unit* to describe these net reactions. Since the result of a transformation can either be a compound of lower molecular weight (MW, e.g., loss of CO_2) or higher MW (e.g., oxidation of alcohols/aldehydes), we differentiate these transformation units into *photo elimination* (product with lower MW) and *photo addition* (product with higher MW).

Without further information, we do not know if the reactions do take place or to which extent and all selected transformations are considered *potential transformations* only. Therefore, next to the commonly applied intensity changes to evaluate phototransformation in DOM, we utilize the temporal information contained in the experiment (a reaction always proceeds forward in time), as well as the intrinsic connection between MF and photochemical pathways (e.g. $C_xH_yO_z$ [educt MF] + hν → $C_{x-1}H_yO_{z-2}$ [product MF] + CO_2, denoted as "*-CO2"*) in order to obtain more detailed information about the presumed reactions (*predicted transformation* in the following) and which molecules are subjected to which transformation.

Implementation of the Graph Model

Neo4j enables efficient data retrieval based on the query language Cypher. Besides manipulating the graph structure (insert, update, delete) users can describe patterns to select matching parts in the graph. The dataset (SI Text Experimental Setup and Data Preparation) is converted into nodes and properties according to the temporal graph model and loaded into the graph database. We used Python (version: 3.10.9) and the library neo4j (version: 5.6.0) to automate the preprocessing (SI Description 1) of the raw files and the creation of the database. The Python package neo4j is an official Neo4j driver and acts as an interface between Neo4j and Python. It allows us to send Cypher queries to the graph database via Python. The initial graph including molecule nodes, "SAME_AS" and "POTENTIAL_TRANSFORMATION" edges (created as described by SI Algorithm 1) is further used to predict the likely occurring chemical transformations (see below).

Calculation of the Weights for "PREDICTED_TRANSFORMATION" Edges

Each "PREDICTED_TRANSFORMATION" edge is further described by a weight (stored as edge property, e.g. see SI Figure 5). This weight is calculated during the link prediction by considering all involved nodes and edges of potential transformations. A "PREDICTED_TRANSFORMATION" edge always starts at a node (n1) with a decreasing intensity trend and ends at a node (n2) with an increasing intensity trend (e.g. Figure 3 step 3). The weight of an edge is composed of two components for increasing and decreasing intensities:

the product of the normalized intensity (*norm_int_incr*) with the intensity trend (*int_trend_incr*) of node n1 with an increasing intensity trend (e.g. Fig. 3 step 3 node A_{t0}).

the product of the normalized intensity (*norm_int_decr*) with the intensity trend (*int_trend_decr*) of node n2 with a decreasing intensity (e.g. Fig. 3 step 3 node C_{t0}). Note that we subtract the trend from 1 to increase the impact of high intensity losses.

The formula to calculate the weight for an "PREDICTED_TRANSFORMATION" edge is:

$$weight = (norm\_int\_incr * int\_trend\_incr) + (norm\_int\_decr * (1 - int\_trend\_decr))$$

The edge weight increases with high increasing or decreasing intensity trends as well as high normalized intensity. A weight of an edge is further normalized (*normalized_weight*) based on the weights of all incoming "PREDICTED_TRANSFORMATION" edges of its end node n2.

Model Validation with Carbamazepine

An additional photo-irradiation experiment was conducted with a model compound of known photochemical reactions. For this, we used Carbamazepine (CBZ) which has well-established photo-products.[9] We use this validation experiment to demonstrate that the "Transformation Prediction Algorithm" (TPA) is

1. accurate. It should be capable of identifying transformations in a time series experiment based on expected peak intensity changes and known transformation units.
2. specific. It should not result in a large number of "false-positive" links between unrelated molecules.

Both aspects are prerequisites to confidently identify transformations in DOM.

Note that due to the non-DOM nature of the compound, a different set of CBZ-TUs was used as compared to the rest of the study, namely "+O", "+H2O2", "+O -CH3N", "+H2O", "-C2H3NO", and "-C2H3NO2", which were derived from the known phototransformation products of CBZ described in the supplement to Raeke et al. (2017).

For the validation experiment, we used the experimental setup of Raeke et al. (2017). 50 µmol L^{-1} of Carbamazepine (99%, Sigma-Aldrich, St. Louis, USA) was dissolved in ultrapure water (Milli-Q, Merck, Darmstadt, Germany). The irradiation experiment was conducted with a solar simulator (Q-SUN Xe-1, Q-Lab, Westlake, USA) equipped with a xenon arc lamp (1800 W) and a Daylight-Q filter (noon summer sunlight). The continuous emission spectra of a xenon arc lamp can closely mimic natural light with a wavelength range of 290 to 800 nm. All experiments were carried out under a radiation intensity of ~780 Wm^{-2}.

Samples were measured in positive mode with the same instrument as the DOM photo-irradiation experiment samples (cf. SI Text Experimental Setup and Data Preparation), accumulating 64 scans at 4 MWord data size and 0.1 s ion accumulation time. Spectra were internally calibrated with CBZ and its known transformation products.[9] Due to the positive ionization, frequent sodium adducts can be observed, resulting in two CBZ species observable in the spectrum: CBZ+H and CBZ+Na. Annotated mass spectra are provided in SI Figure 16.

With the data from the validation samples, a new temporal graph was constructed and the TPA used to identify and link CBZ with its photo-products according to the CBZ-TU list (SI Figures 17-19). Besides the two CBZ species, only 3 other molecules present in the sample were identified by the TPA, although the CBZ-TU list also contains common DOM photo-transformation units like "+O" or "+H2O" (see SI Table 1). It needs to be noted that unlike typical DOM samples, some CBZ photo products may not be present in the original sample. The TPA requires that a molecule is present in two consecutive snapshots, in order to calculate the intensity trend as an important consideration for the transformation identification (cf. SI Description 2).

The outcome of the validation experiment confirms, that the TPA

1. is able to reliably extract the information from the known CBZ phototransformation pathways from the temporal graph,
2. has only a small "false-positive rate" despite non-specific TUs due to the importance of the intensity change consideration during the identification.

SI Descriptions

SI Description 1. Preprocessing steps to create the temporal graph from separated files.

- Create a metadata file with information about the snapshots, radiation doses and measurement ids. The measurement ids need to be mapped to the measurement ids of the file with the molecules.
- Clean the file with the actual molecules. The cleaning includes for example replacing all non-existing numeric values with zero, extracting the needed columns for the graph model and creating a separate file with unique molecular formulas.
- Calculate the potential chemical transformations between every pair of two MF (SI Algorithm 1) from the unique molecular formulas based on common photochemical reactions (SI Table 1).

SI Description 2. Considerations used in the "Transformation Prediction Algorithm" to calculate the "PREDICTED_TRANSFORMATION" edges based on intensity trends of molecules in the graph model.

Consid. 1: If the intensity of molecules with the same molecular formula A_{tx} and A_{tx+1} increases from snapshot t_x to t_{x+1}, the chemical transformations of molecules from snapshot t_x to molecule A_{tx+1} are weighted higher than the chemical transformations from molecule A_{tx} to molecules in snapshot t_{x+1}. Therefore, outgoing potential chemical transformations edges from the start node A_{tx} of the "SAME_AS" edge are excluded and incoming edges to the end node A_{tx+1} of the "SAME_AS" edge are included.

Consid. 2: If the intensity of molecules with the same molecular formula A_{tx} and A_{tx+1} decreases from snapshot t_x to t_{x+1}, the chemical transformations of molecules from snapshot t_x to molecule A_{tx+1} are weighted lower than chemical transformations from molecule A_{tx} to molecules in snapshot t_{x+1}. Therefore outgoing potential chemical transformation edges from the start node A_{tx} of the "SAME_AS" edge are included and incoming edges to the end node A_{tx+1} of the "SAME_AS" edge are excluded.

Consid. 3: If the intensity of a molecule is below a threshold, the molecule has a balancing number of chemical transformations. Therefore, the incoming and outgoing potential chemical transformation edges are excluded.

SI Description 3. Iterative steps implemented in the "Transformation Prediction Algorithm" to calculate the "PREDICTED_TRANSFORMATION" edges based on intensity trends of molecules in the graph model (cf. Figure 3 in the main text, SI Algorithm 2).

- In step 1 the algorithm checks if the node A_{tx+1} at snapshot t_{x+1} has an incoming "SAME_AS" edge from snapshot t_x. If node A_{tx+1} does not have an incoming "SAME_AS" edge the algorithm selects the next node and starts again. The algorithm moves to step 2 if the intensity trend at the "SAME_AS" edge increases.

- In step 2 the algorithm looks for all "POTENTIAL_TRANSFORMATION" edges to node A_{tx+1} in snapshot t_{x+1}. The algorithm collects the nodes at the start of the "POTENTIAL_TRANSFORMATION" edges from snapshot t_x. Every collected node with an increasing or consistent intensity trend at the "SAME_AS" edge to snapshot t_{x+1} will be removed from the collection.

- In step 3 only nodes with a decreasing intensity trend are still in the collection. The remaining "POTENTIAL_TRANSFORMATION" edges from snapshot t_x to node A_{tx+1} describe the predicted chemical transformations and will be saved in the graph as "PREDICTED_TRANSFORMATION" edges.

SI Algorithms

SI Algorithm 1. Calculation of potential chemical transformations

```
1.  Input: Set of molecules M, Set of transformation units TU
2.  Output: Set of potential transformations PoT
3.
4.  PoT ← ∅
5.  for m ∈ M
6.    for tu ∈ TU
7.      // generate PoT based on addition
8.      if tu.isRelevantAddition() then
9.        moleculeAddition ← calcSum(m.getAtoms(), tu.getAtoms())
10.       if M.has(moleculeAddition) then
11.         PoT ← addPair(m, moleculeAddition)
12.     // generate PoT based on elimination
13.     if tu.isRelevantElimination() then
14.       moleculeElimination ← calcDiff(m.getAtoms(), tu.getAtoms())
15.       if M.has(moleculeElimination) then
16.         PoT ← addPair(m, moleculeElimination)
17. return PoT
```

We now describe the single steps for SI Algorithm 1 to calculate potential chemical transformations (PoT). The input is a set of molecules **M** (obtained from the molecule data we want to analyze) and the predefined set of transformation units **TU** including their assessment of relevance in direction photo addition and elimination (line 1). The output is the set of potential chemical transformations **PoT** (line 2). Initially, we define an empty set for the calculated potential chemical transformations **PoT** (line 4). We iterate through the given set of molecules **M** (line 5) and for each molecule **m**, we then iterate through the given set of transformation units **TU** (line 6). For every molecule **m** and transformation unit **tu**, we check whether the current transformation unit **tu** is marked as relevant for addition in the predefined set of transformation units **TU** (line 7-8). If yes, we calculate the sum of the atoms of the current molecular formula **m** and the current transformation unit **tu** (line 9) to obtain a candidate **moleculeAddition**. We then check if the candidate molecular formula exists in the set of all considered molecules **M** (line 10). If yes, we add the current molecular formula **m** and the new calculated molecular formula **moleculeAddition** as a new pair to the set of potential chemical transformations **PoT** (line 11). We follow the same process in the direction of elimination for the same molecule **m** and transformation unit **tu**. We check if the current transformation unit **tu** is marked as relevant for elimination in the predefined set of transformation units **TU** (line 12-13), calculate this time the difference of the atoms of the current molecular formula **m** and transformation unit **tu** (line 14) and finally check the existence of the calculated molecular formula **moleculeElimination** in the set of all considered molecules **M** (line 15). If so, we add the pair of the current molecular formula **m** and the new calculated molecular formula **moleculeElimination** to the set of potential chemical transformation **PoT** (line 16). The process is

repeated until all molecules in **M** have been visited (line 5). As a result, we return the obtained set of the calculated potential chemical transformations **PoT** (line 17).

Note, that the output of algorithm 1 is used to create an initial version of the temporal graph. First, we add the set of molecules in different snapshots as nodes N to the graph G. The nodes in subsequent snapshots are then interconnected by a set of edges E of two different types: SAME_AS edges and the PoT edges (output of SI Algorithm 1). This graph is the main input for SI Algorithm 2.

SI Algorithm 2. Transformation Prediction Algorithm to predict the likely occurring chemical transformations

```
1.  Input: Graph G with nodes N and edges E, margin of error e
2.  Output: Graph G with added edges of type ‘PREDICTED_TRANSFORMATION’ (typePrT)
3.
4.  typePoT ← ’POTENTIAL_TRANSFORMATION’
5.  typeSaS ← ’SAME_AS’
6.  e ∈ [0,1]
7.  for n ∈ N
8.    // get intensity trend from preceding snapshot of node n (Figure 3, step 1)
9.    intTrend ← getIntensityTrend(n.getIncomingEdges(typeSaS))
10.   if intTrend > 1 + (0.5 * e) then
11.     // collect all nodes with a potential transformation edge to n (Figure 3, step 2)
12.     N’ ← getStartNode(n.getIncomingEdges(typePoT))
13.     for n’ ∈ N’
14.       // get intensity trend of node n’ to succeeding snapshot (Figure 3, step 3)
15.       intTrend’ ← getIntensityTrend(n’.getOutgoingEdges(typeSaS))
16.       if intTrend’ < 1 - (0.5 * e) then
17.         G ← addEdgetypePrT(n’, n)
18. return G
```

We now describe the single steps for SI Algorithm 2. The input is graph **G** consisting of nodes **N** and edges **E** (SI Algorithm 1) as well as the defined margin of error **e** (line 1). As output, we want the graph **G** with the added predicted likely occurring chemical transformation edges **PrT** (line 2). We define two variables for the edge types **typePoT/typeSaS** to increase readability of the pseudocode (line 4-5). The margin of error **e** defines the inaccuracy in the data and is set to a value between 0 and 1 (line 6) (typically 5 % / 0.05). Intensity trends **intTrend** above 1+(0.5*e) are considered as increasing while trends below 1-(0.5*e) are considered as decreasing. For each node **n** in the graph **G** (line 7), we check the intensity trend **intTrend** of the current node **n** from the preceding snapshot to the current snapshot along the “SAME_AS” edge **typeSaS** (line 8-9) and continue only if the intensity trend **intTrend** is considered as increasing (line 10). We then collect all nodes **n’** with a “POTENTIAL_TRANSFORMATION” edge **typePoT** to the current node **n** (line 11-12). For each collected node **n’** (line 13), we check the intensity trend **intTrend** from their current snapshot to the succeeding snapshot along their “SAME_AS” edge **typeSaS** (line 14-15). If the intensity trend **intTrend** is considered as decreasing (line 16), we add a

"PREDICTED_TRANSFORMATION" edge **typePrT** from the remaining collected nodes **n'** to the currently observed node **n** (line 17). The process is repeated until all nodes **n** have been viewed (line 7). As a result, we return the graph **G** with all added predicted likely occurring chemical transformations (edge **typePrT**) (line 18).

SI Tables

SI Table 1. Transformation units considered in the calculation of the "POTENTIAL_TRANSFORMATION" edges, divided into *photo addition* (green) and *photo elimination* (orange) based on the net change in molecular mass. The selection was adapted from Hu et al. (2022).[2]

Transformation Unit [Formula Difference]	Transformation Group	Mass Difference	Chemical Reaction	Reference/ Comment
+O-NH	addition	+0.984	deamination	[2,3]
-O+NH	elimination	-0.984	amination	[4]
+O-CH2	addition	+1.979	oxidation + terminal C cleavage	#
-H2	elimination	-2.016	oxidation: alcohol -> ketone/aldehyde	[5,6]
-C	elimination	-12	chain splitting	#
+O-H2	addition	+13.979	oxidation: alcohol -> acid	[7]
-CH2	elimination	-14.016	chain splitting	[2], #
+O	addition	+15.995	oxidation: aldehyde -> acid	[7]
+H2O	addition	+18.011	hydration	[8,9]
-H2O	elimination	-18.011	dehydration	[10]
+O2-H4	addition	+4.032	multi-oxidation	[7]
-CO	elimination	-27.995	decarbonylation	[11]
-C2H2	elimination	-28.031	chain splitting	[2], #
-S	elimination	-31.972	desulfonylation	[12], #
+O2	addition	-31.990	multi-oxidation	[7]
-CO2	elimination	-43.99	decarboxylation	[13]
-C2H4O	elimination	-44.026	deacetylation	
+NO2-H	addition	+44.985	nitration	[8]
-NO2+H	elimination	-44.985	denitration	[14]
-SO	elimination	-47.967	desulfonylation	[12], #
-C4H4O	elimination	-56.026	deacetylation	
-SO3	elimination	-79.957	desulfonylation	[15,16]

requires addtl. reaction steps

SI Table 2. Summary of the first four clusters derived from the *label propagation algorithm*. Cluster color code according to Figure 7 in the main text.

Cluster	# MF	CHNO	CHO	CHOS	Mean Normalized Intensity	Mean Intensity Trend	Mean Potential Transformation Edges	Mean Predicted Transformation Edges
1	1945	1060	796	89	0.98	1.04	10.6	1.49
2	560	49	472	39	1.04	1.57	9.97	1.39
3	436	2	430	4	0.95	0.94	10.2	1.37
4	393	0	393	0	0.98	1.25	9.36	1.27

SI Table 3. Mean molecular descriptors of the MF in the first four clusters derived from the *label propagation algorithm*. Cluster color code according to Figure 7 in the main text.

Cluster	Mean H/C	Mean O/C	Mean N/C	Mean Mass	Mean AI$_{mod}$	Mean NOSC	Mean DBE	Mean DBE-O
1	1.036	0.437	0.043	341	0.409	-0.025	9.3	2.2
2	1.573	0.386	0.005	445	0.095	-0.776	6.1	-2.2
3	0.942	0.428	0.000	547	0.449	-0.085	15.5	3.7
4	1.250	0.487	0.000	592	0.221	-0.276	11.5	-1.9

SI Table 4. Transformation units and their average share (in %) in the first four clusters (and their mean) derived from the *label propagation algorithm*. The transformation units are colored based on their attribution to the *photo addition* (green) or *photo elimination* (orange) group. Cluster color code according to Figure 7 in the main text.

Transformation Unit	Cluster 1	Cluster 2	Cluster 3	Cluster 4	Mean
-C1 -H2	6.0	7.4	7.9	8.4	7.4
H2 O1	6.4	8.6	6.4	7.6	7.2
-C1	6.4	7.7	6.8	7.7	7.2
-C1 -O1	6.4	7.1	7.9	7.3	7.2
O1	6.0	7.0	7.1	8.2	7.1
-C2 -H2	6.2	7.9	5.5	7.3	6.7
-H2	5.8	5.4	7.6	6.9	6.4
-C1 -H2 O1	5.4	6.2	6.7	7.3	6.4
O2	5.2	7.1	6.4	6.5	6.3
-H2 O1	5.4	4.5	8.2	7.1	6.3
-C1 -O2	5.7	6.3	6.8	4.6	5.9
-C2 -H4 -O1	5.7	5.2	6.0	5.1	5.5
-C4 -H4 -O1	5.4	8.2	3.0	5.2	5.4
-H2 -O1	4.9	4.4	5.8	5.5	5.1
-H4 O2	4.1	3.3	7.8	5.2	5.1
H1 -O1 N1	4.4	0.1	0.0	0.0	1.1
-H1 O2 N1	4.0	0.0	0.1	0.0	1.0
H1 -O2 -N1	3.1	1.0	0.0	0.0	1.0
-H1 O1 -N1	2.9	0.9	0.0	0.0	1.0
-S1	0.3	0.8	0.1	0.0	0.3
-O1 -S1	0.4	0.5	0.0	0.0	0.2
-O3 -S1	0.2	0.4	0.0	0.0	0.2

SI Figures

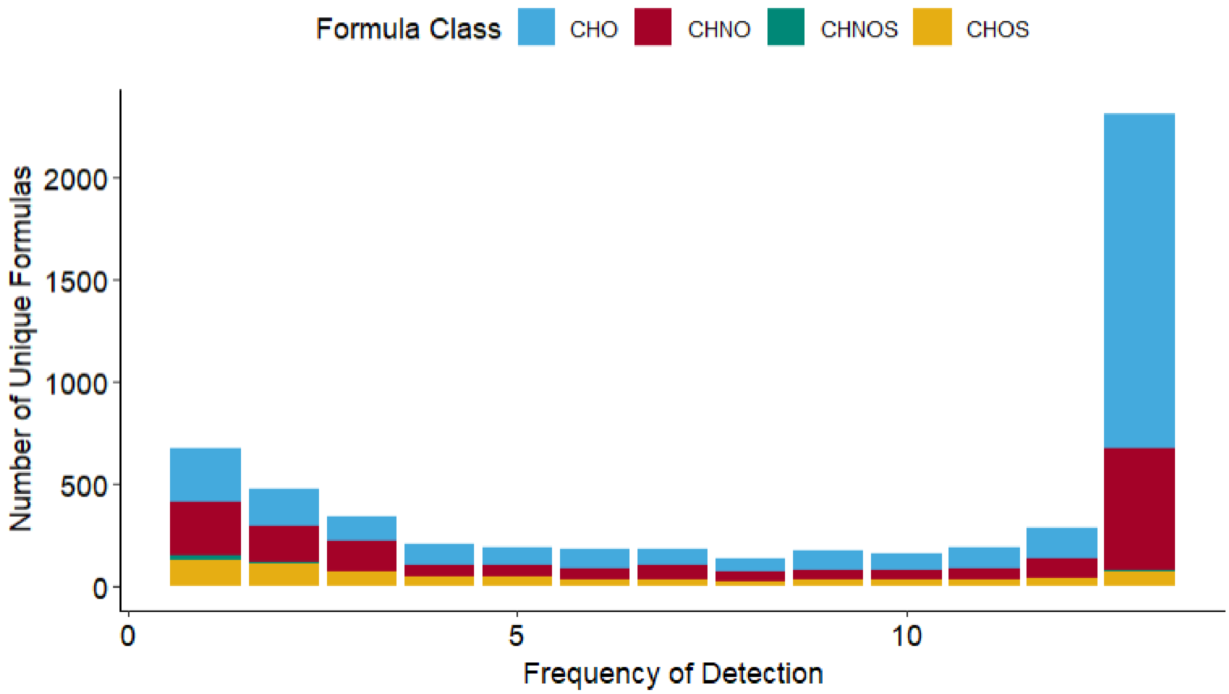

SI Figure 1. Distribution of unique MF (n = 5521) detected in the data set according to their frequency of detection. Color code according to the main formula classes CHO (blue), CHNO (red), CHNOS (cyan), and CHOS (yellow).

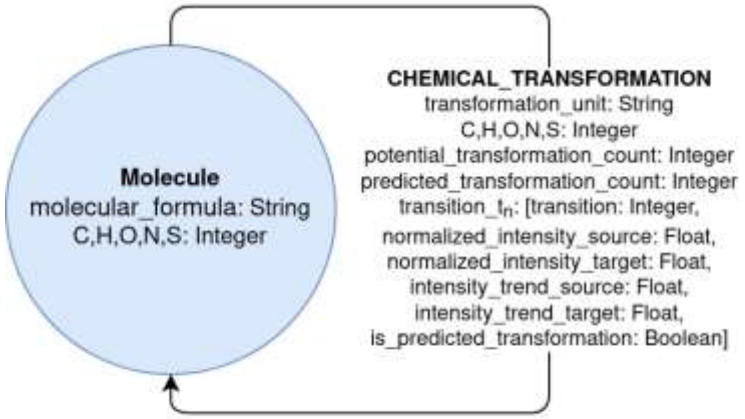

SI Figure 2. The *light temporal graph* model. The nodes contain only static properties (e.g. molecular formula). All edges of the temporal graph model (cf. Figure 1 in the main text) are reduced to one edge type, “CHEMICAL_TRANSFORMATION”. The temporal properties of all snapshots in the temporal graph are stored as separate lists in the properties of type “transition_t_n”. The occurrence of a molecule at a specific time/snapshot can still be identified with the “CHEMICAL_TRANSFORMATION” properties.

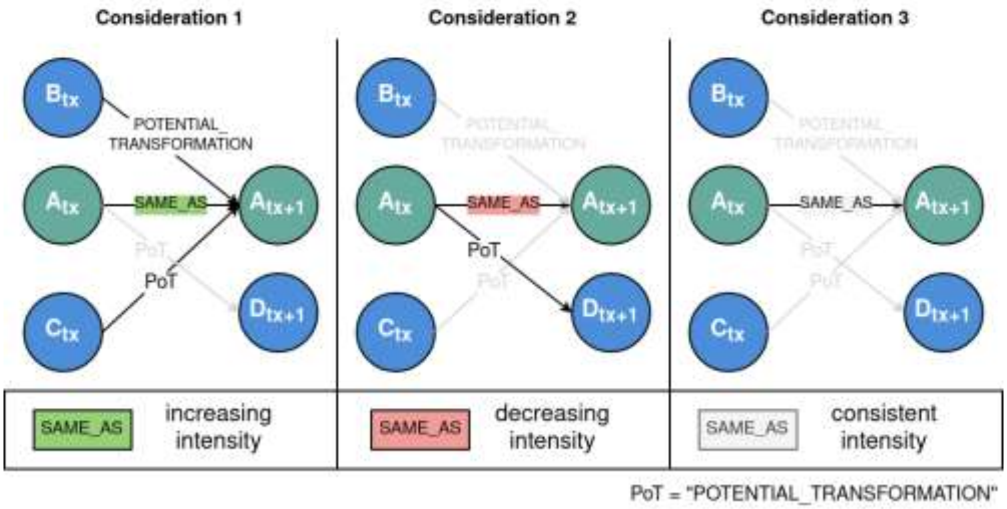

SI Figure 3. The three considerations implemented by the "Transformation Prediction Algorithm". Cf. SI Description 2 for a more detailed description of the considerations.

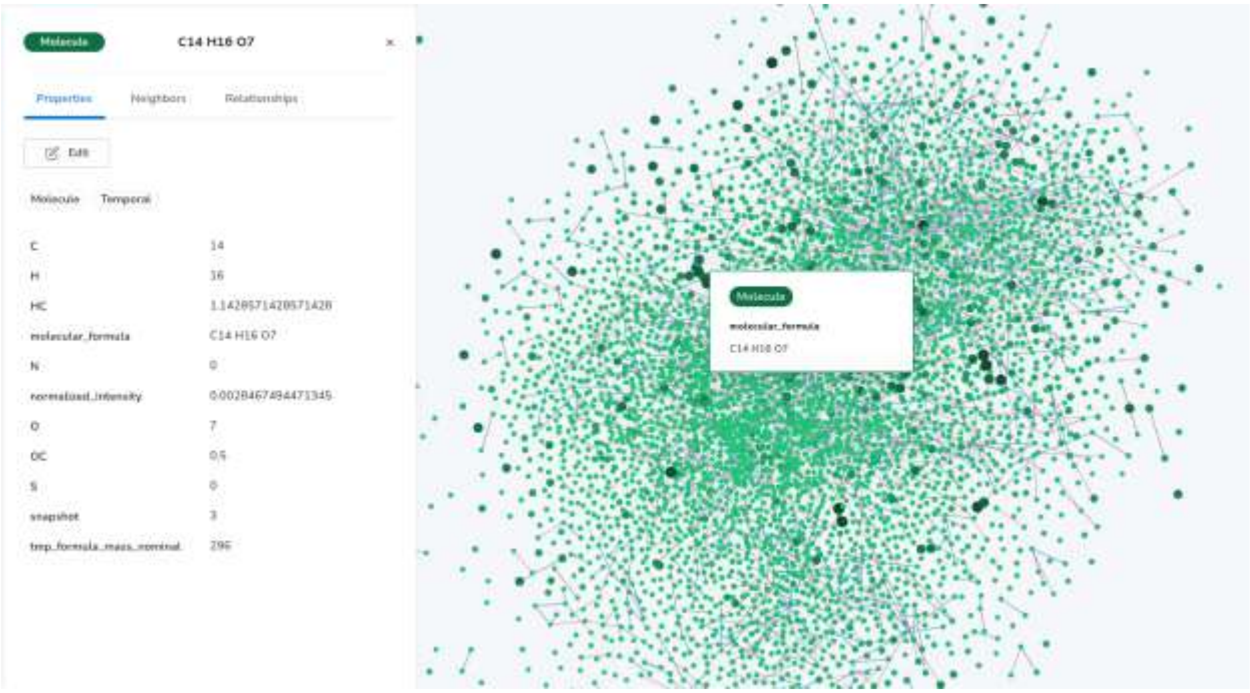

SI Figure 4. Small extract of the created temporal graph with nodes describing the molecules and edges describing the predicted likely occurring chemical transformations. The properties of the highlighted molecule are shown on the left. The edges of some of the occurring chemical transformations are coded in a different color ("H2O", "-C", "-CO"). Darker and larger nodes have a higher normalized intensity.

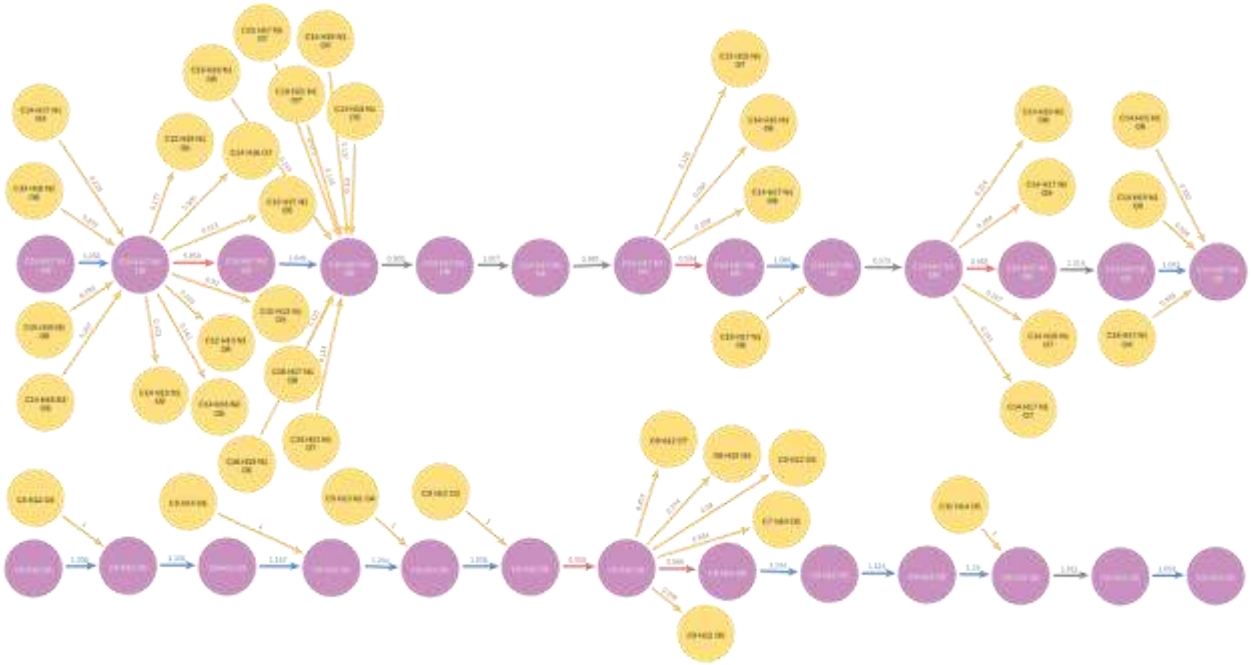

SI Figure 5. Visualization of the sub-graph for MF (A) “C14 H17 N1 O6” and (B) “C9 H12 O5” with incoming and outgoing *predicted transformations* across all snapshots. Arrows indicate the “SAME_AS” edges with the intensity trend (blue=increasing, gray=consistent, red=decreasing) and the “PREDICTED_TRANSFORMATION” edges with their weight (orange). The weight at the incoming “PREDICTED_TRANSFORMATION” edges shows the relative contribution of the respective transformation unit (not shown) to the increasing intensity at the “SAME_AS” edge. The weight at the outgoing “PREDICTED_TRANSFORMATION” edges show the influence of the molecule to the intensity development of the end nodes (i.e., the product of the transformation).

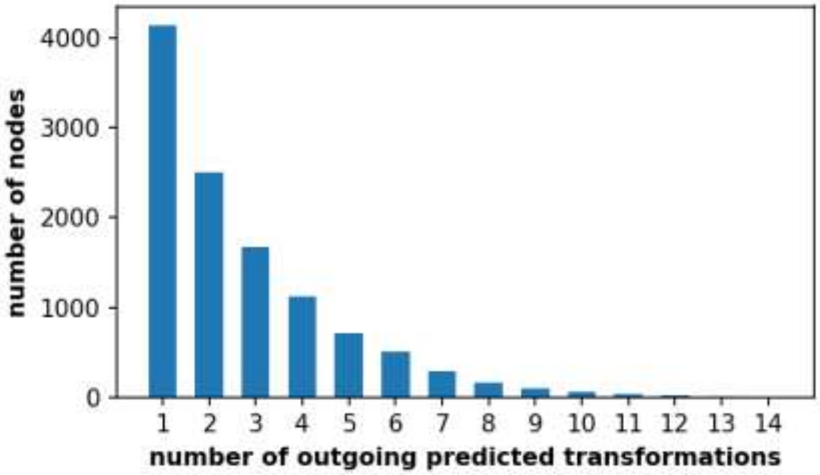

SI Figure 6. Number of outgoing predicted transformations per node. Most nodes have only a few outgoing predicted transformations and a few nodes have many outgoing predicted transformations. A common pattern in scale-free networks.

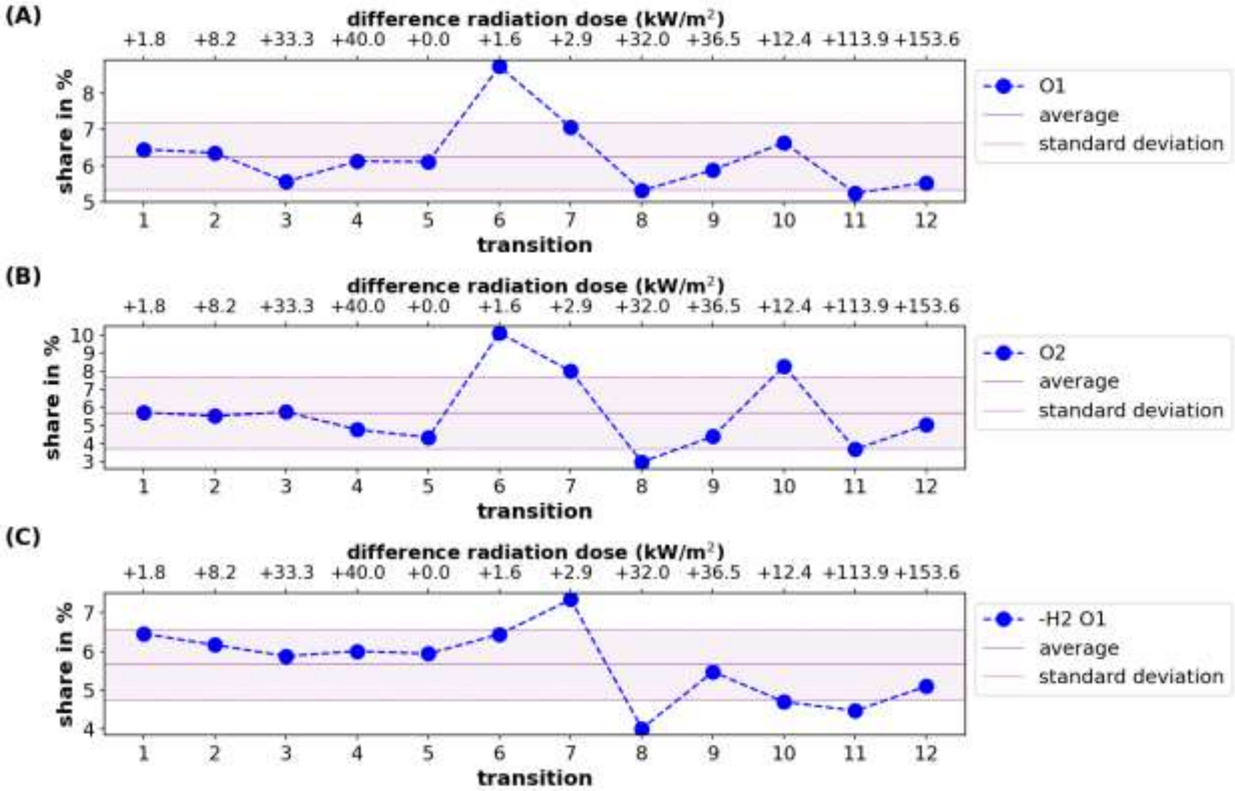

SI Figure 7. Selected transformation units associated with photo-oxidation reactions and the development of their share over time. (A) Transformation unit “+O” (B) Transformation unit “+O2” (C) Transformation unit “-H2+O”.

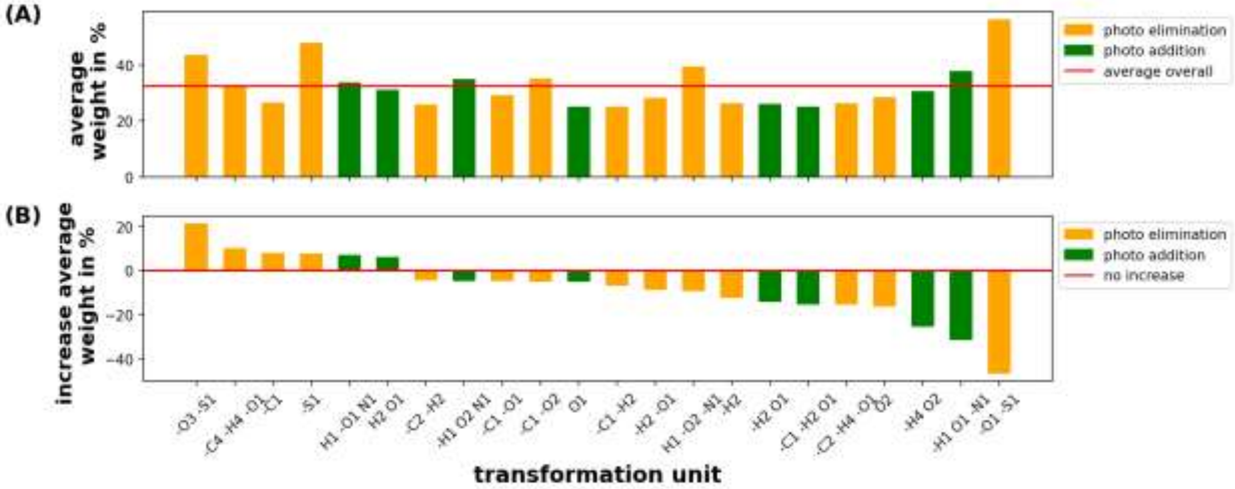

SI Figure 8. (A) Calculated normalized average weight of the transformation units at the “PREDICTED_TRANSFORMATION” edges and (B) their trend over the experimental time course. The weight was calculated using the intensity trends at the “SAME_AS” edges and the normalized intensity of the nodes. The weight increases with a high intensity trend and a high normalized intensity. The incoming "PREDICTED_TRANSFORMATION" edges of each node were normalized based on the calculated weights so that they sum to 100%. The higher the average weight the more influence the transformation unit had.

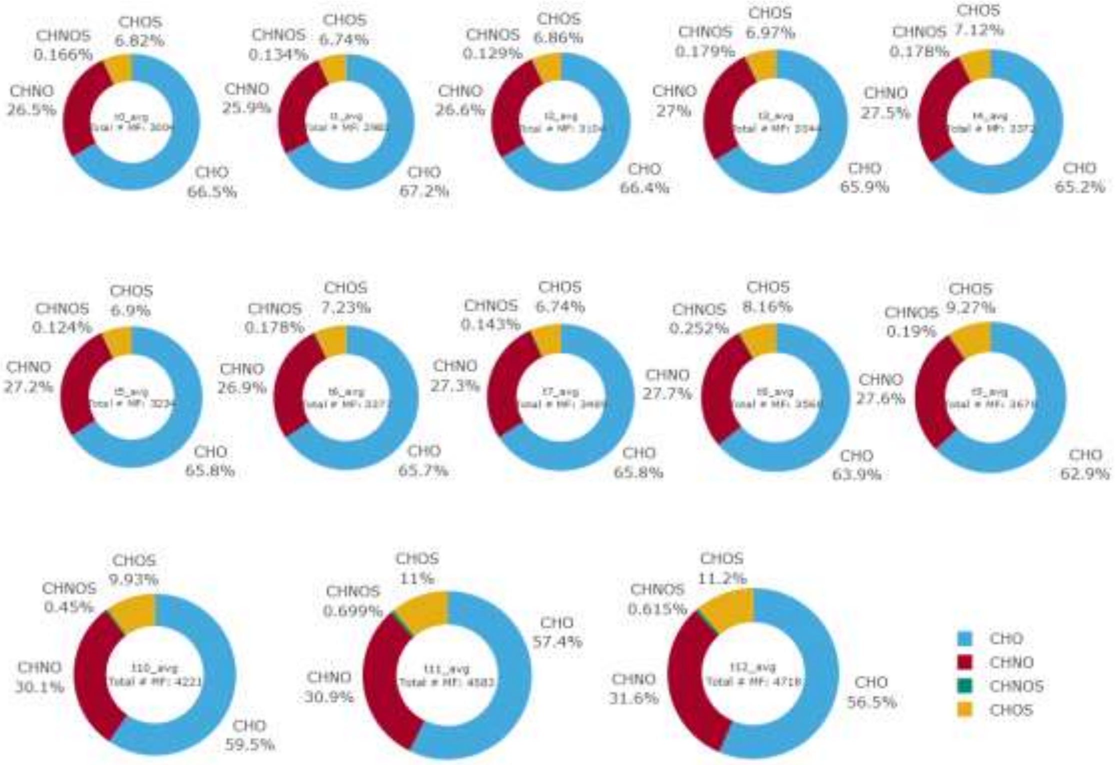

SI Figure 9. Formula class distribution for all samples in the photo experiment data set. Color code according to the main formula classes CHO (blue), CHNO (red), CHNOS (cyan), and CHOS (yellow).

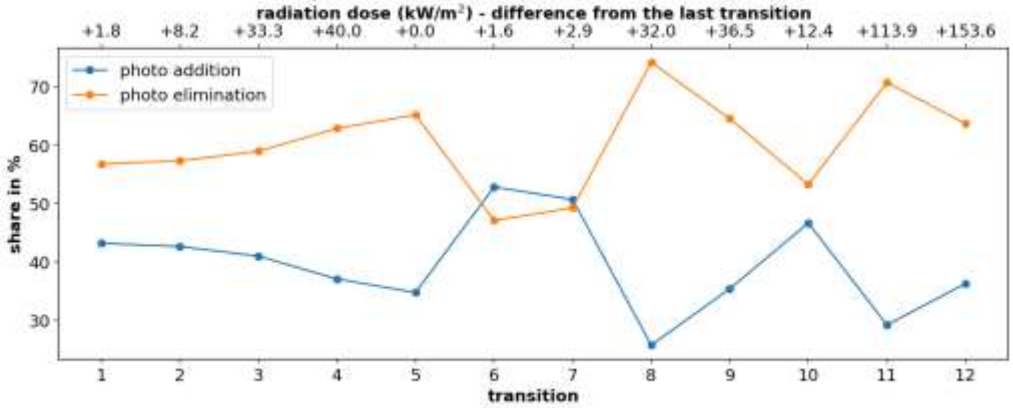

SI Figure 10. Summed share of *photo addition* and *photo elimination* processes over time (cf. SI Table 1).

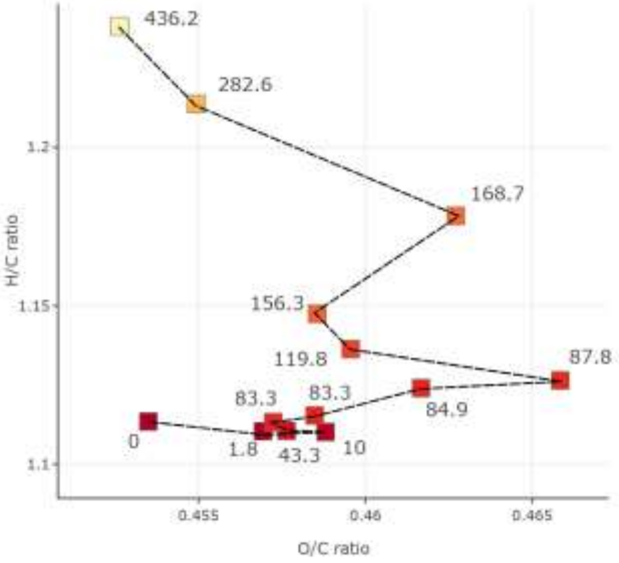

SI Figure 11. Intensity weighted average molecular descriptors H/C vs O/C for all 13 samples in the photo experiment data set. Color corresponds to accumulated radiation dose with actual values provided in kW/m², cf. Wilske et al. (2020). The dashed line is added as visual aid to represent the time course of the experiment.

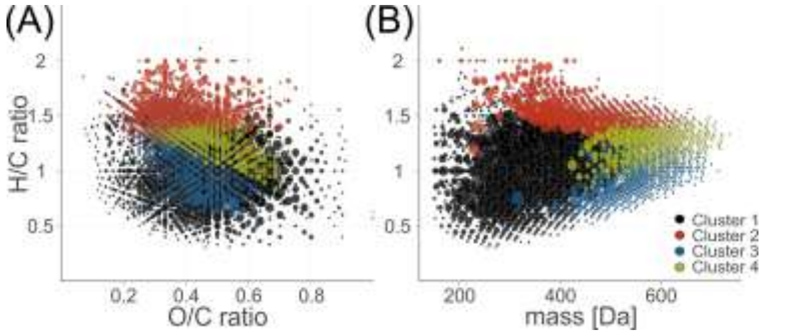

SI Figure 12. Community detection with the *Label Propagation Algorithm* of MF with similar transformations in the photodegradation experiment. The top four clusters cover 62% of all MF in the data set (n = 5521) and are displayed as (A) molecular H/C versus O/C ratio and (B) molecular H/C versus molecular mass. The size of the circles indicates the mean normalized intensity of the respective MF in the data set.

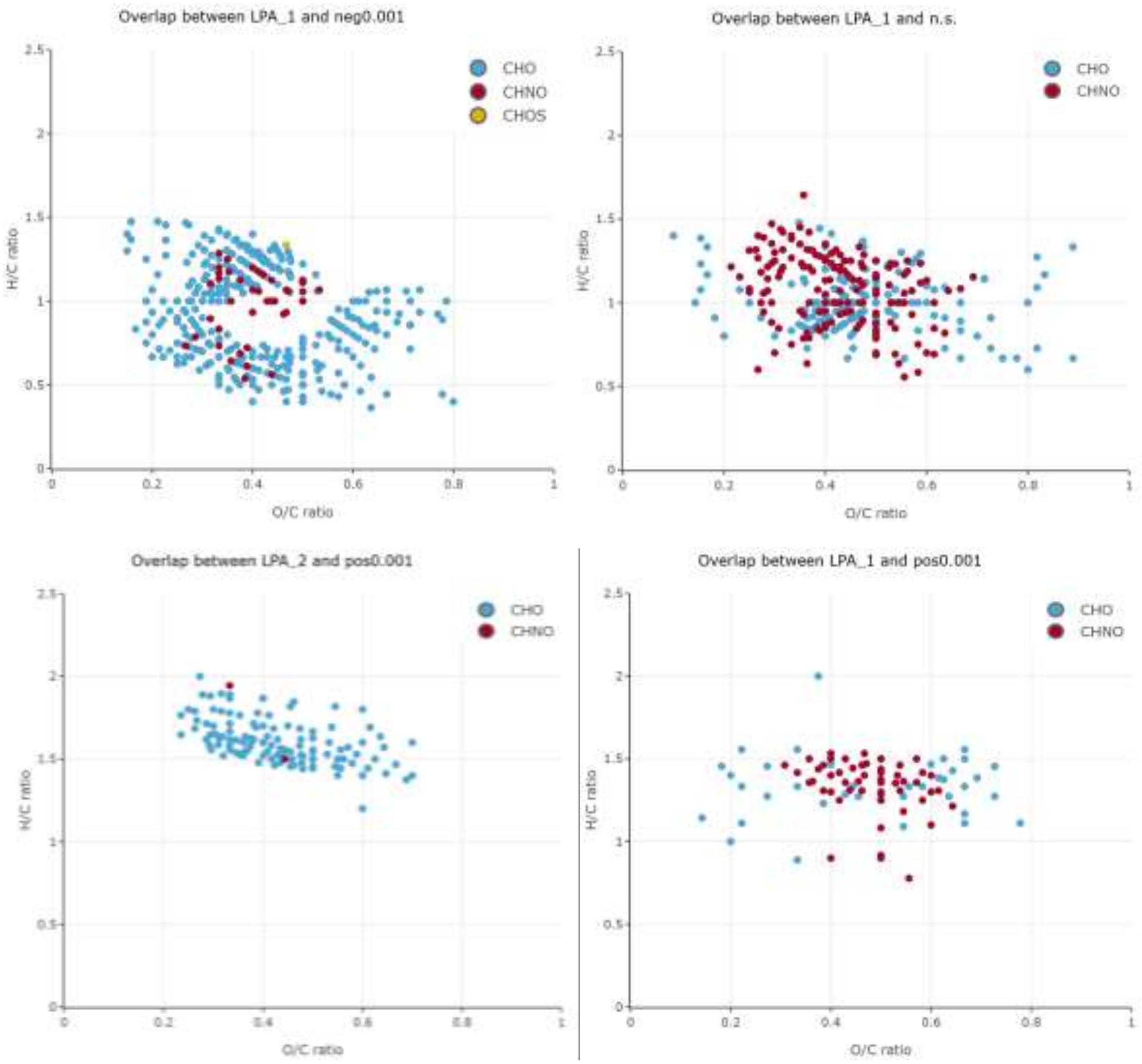

SI Figure 13. Comparison of MF in the first two clusters (LPA_1 and LPA_2) from the *label propagation algorithm* with the highly significant MF (neg0.001 and pos0.001) derived from photoreactivity evaluation from Wilske et al. (2020). Note that neg and pos describe whether a MF is considered degraded (neg) or a photoproduct (pos). The non-significant (n.s.) MF from Wilske et al. (2020) were included as well, since they represent the second largest overlap, cf. SI Figure 14. Color code according to the main formula classes CHO (blue), CHNO (red).

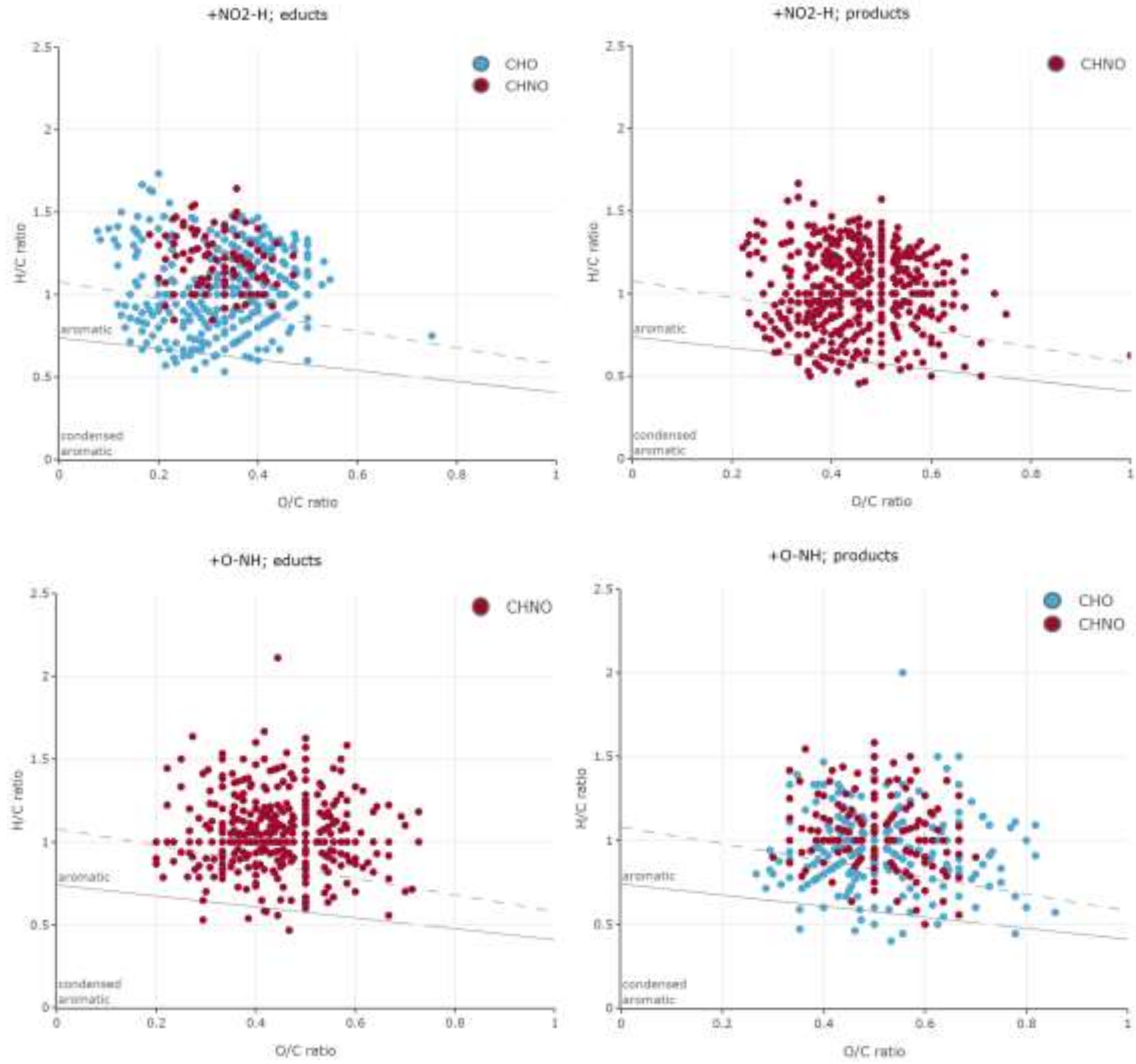

SI Figure 14. MF detected as educts (left) and products (right) for the transformation units "+NO2-H" (top) and "+O-NH" (bottom) within the first cluster (LPA_1) of the *label propagation algorithm*. Color code according to the main formula classes CHO (blue), CHNO (red).

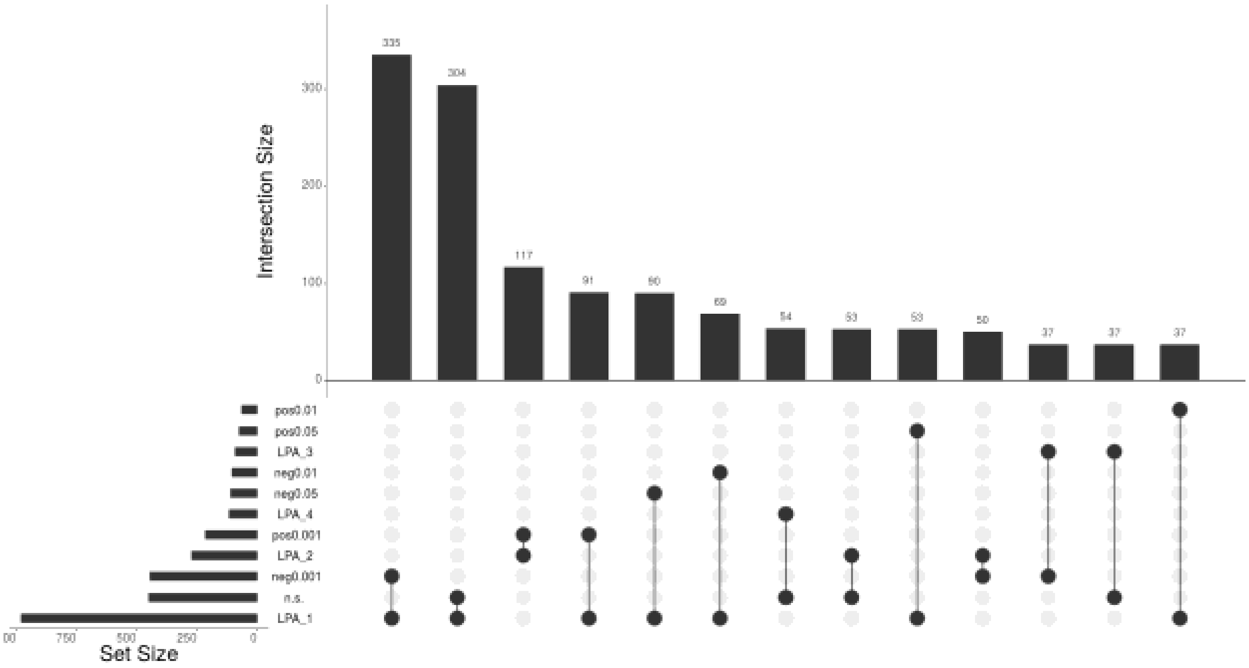

SI Figure 15. Comparison of MF in the four main clusters from the *label propagation algorithm* with the photoreactivity evaluation from Wilske et al. (2020). The clusters are labeled LPA_X and the results from the rank correlation (peak intensity vs DOC concentration) are labeled according to the groups in Wilske et al. (2020). E.g., "neg0.001" means the negatively correlating MF at a significance level of 0.001. Note that due to differences in the data pre-processing, the set size for both data evaluations are different (n = 1999 in Wilske et al. (2020) vs n = 3391 here). Cf. SI Table 2 and SI Table 3 regarding cluster properties and average molecular descriptors.

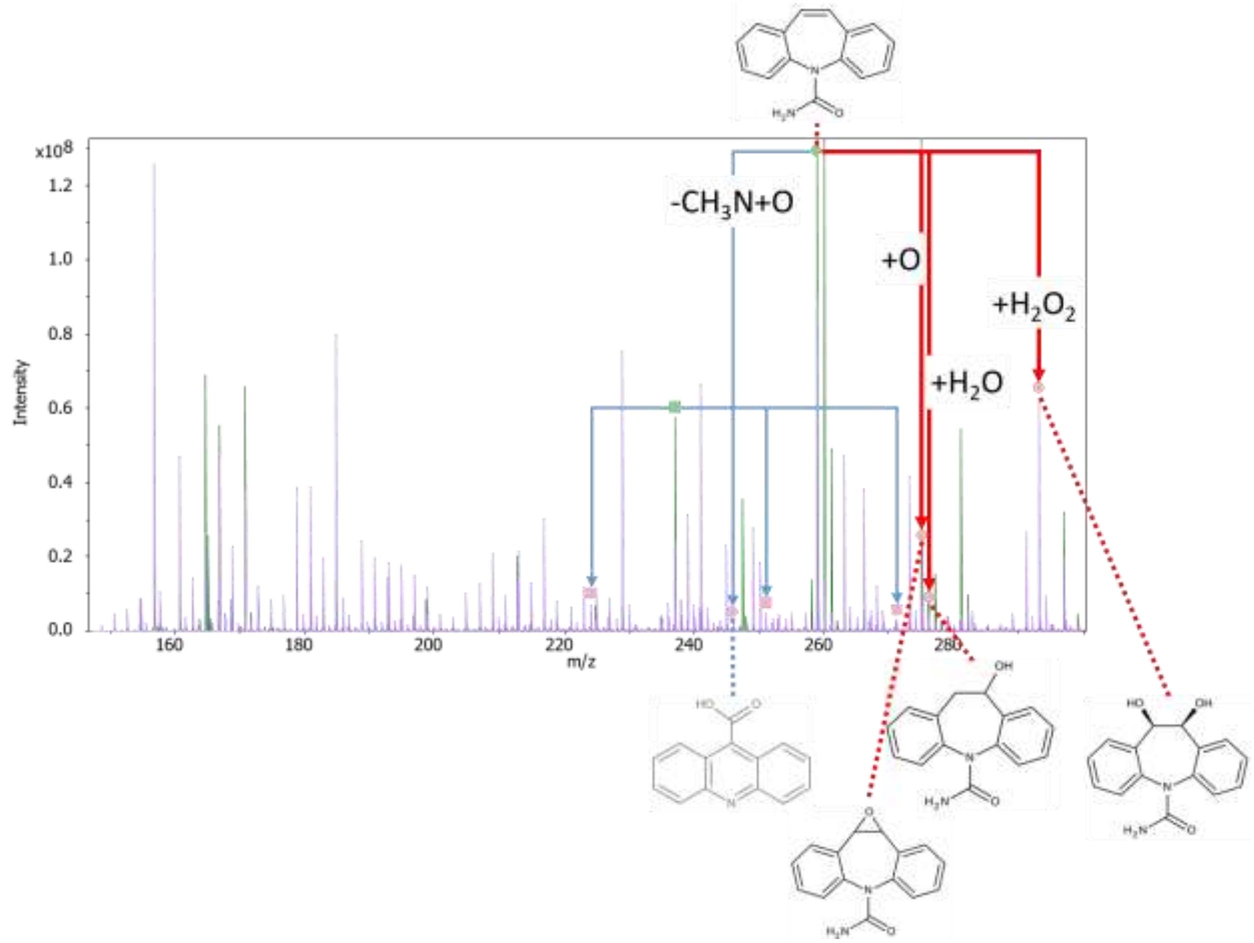

SI Figure 16. FT-ICR mass spectra of 50 µmol L^{-1} Carbamazepine (CBZ) before (green) and after (purple) photoirradiation. Both the protonated (CBZ+H; green square) and the sodiated (CBZ+Na; green circle) form were detected and the structure of CBZ is shown above the $[M+Na]^{+}$ peak at m/z 259.0842. The transformations of the CBZ+Na are indicated with red arrows and the respective transformation units (TU, cf. SI Text Model Validation with Carbamazepine), the CBZ+H transformations in blue. Transformation products of CBZ+Na, which were identified by the "Transformation Prediction Algorithm" (TPA), are indicated by purple circles, and their structures are provided below the mass spectrum. Note that the intensity scale of the spectrum before irradiation is cut at $1.3x10^8$ due to the very intense CBZ+Na peak. Also, Acridine-9-carboxylic acid (m/z 224.070605 $[M+Na]^{+}$, TU: "$-CH_3N+O$") was not identified by the TPA as transformation product, since no peak with the same m/z value was detected in the spectrum before irradiation (cf. SI Text Model Validation with Carbamazepine).

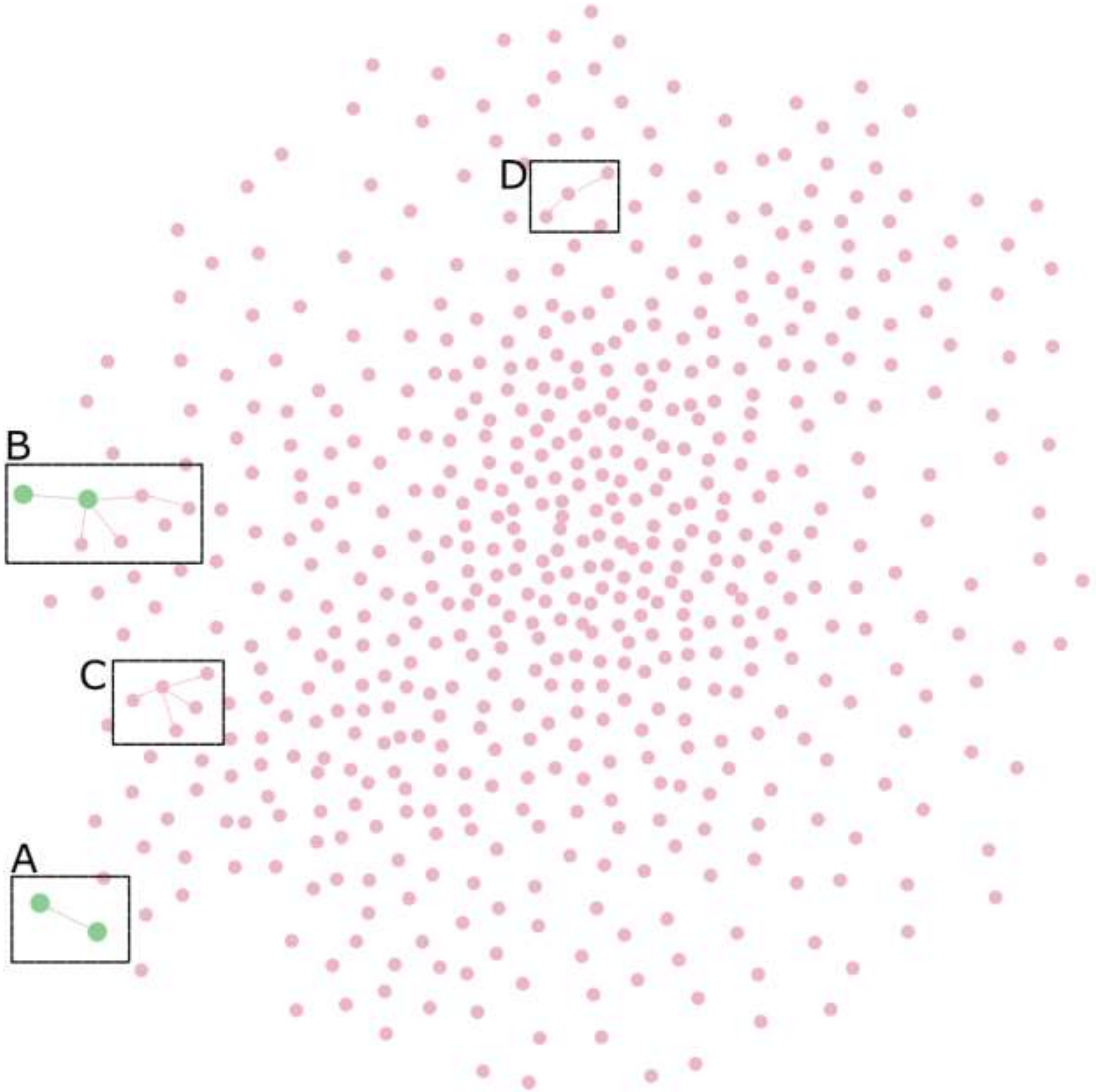

SI Figure 17. Visualization of the Carbamazepine graph to validate the approach. Two consecutive snapshots (before and after photoirradiation, cf. SI Text Model Validation with Carbamazepine) are used for this validation. The green nodes present Carbamazepine with and without sodium (boxes A and B, see SI Figure 18 for expanded view on these subgraphs) and are connected via the "SAME_AS" edges across snapshots. Other displayed edges show the predicted, likely occurring chemical transformations ("PREDICTED_TRANSFORMATION") in this sample. All other nodes without edges do not have "PREDICTED_TRANSFORMATION" edges. Based on the dedicated set of transformation units, two other molecules in the sample were found by the "Transformation Prediction Algorithm" (TPA) having the same transformation units (CBZ-TUs) as Carbamazepine (Boxes C and D, see SI Figure 19 for expanded view on these subgraphs).

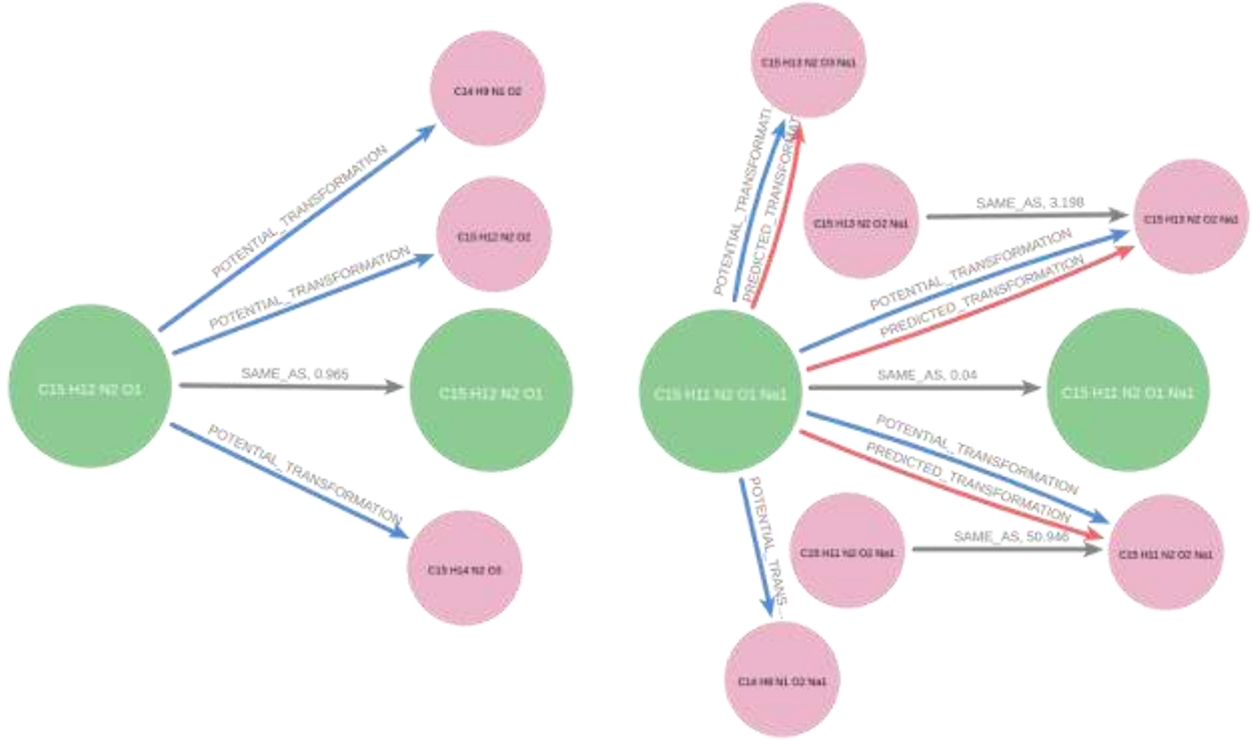

SI Figure 18. Expanded view of the Carbamazepine subgraphs (Boxes A and B in SI Figure 17). The Carbamazepine species without sodium (Box A: CBZ+H; left) decreased in intensity due to photoirradiation, indicated by the intensity trend at the gray arrow ("SAME_AS" edges). The "POTENTIAL_TRANSFORMATION" edges (blue) indicate the possible chemical transformations based on the dedicated set of transformation units (CBZ-TUs, cf. SI Text Model Validation with Carbamazepine). The molecules at the end of the edges were not detected in the initial sample and are therefore not considered by the "Transformation Prediction Algorithm" (TPA). The Carbamazepine molecule with sodium (Box B: CBZ+Na; right) largely decreased (very small intensity trend value at the "SAME_AS" edges). In this case, some molecules at the end of the "POTENTIAL_TRANSFORMATION" edges already exist in the first snapshot and can therefore be considered by the TPA (two examples, C15 H11 N2 O2 Na1 and C15 H13 N2 O2 Na1, are shown with their respective "SAME_AS" edges and intensity trends). Consequently, the predicted, likely occurring chemical transformations ("PREDICTED_TRANSFORMATION") are indicated by the red arrows (cf. red arrows in SI Figure 16). Those "PREDICTED_TRANSFORMATION" edges subsequently used in the temporal graph analysis steps (cf. Figure 4 and 5 in the main text). Note that for the CBZ+Na subgraph, all molecules contain Na, since the considered CBZ-TUs do not involve Na.

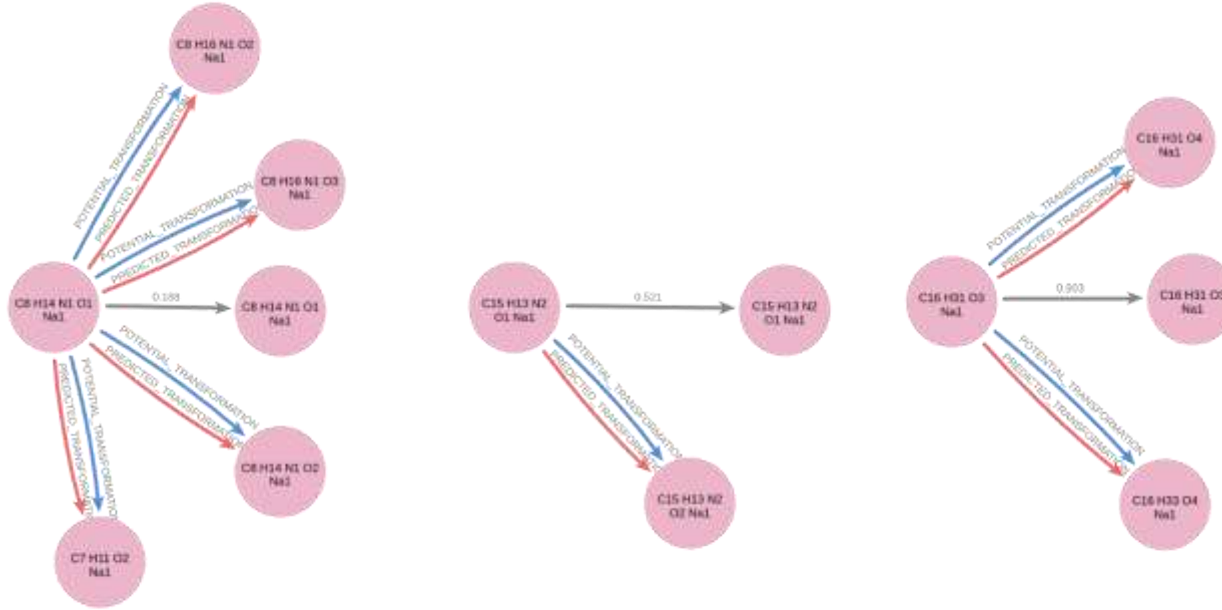

SI Figure 19. Expanded view of the subgraphs of molecules which have predicted, likely occurring chemical transformations besides carbamazepine (SI Figure 18). Some other molecules in the sample also fulfill the conditions to be considered by the "Transformation Prediction Algorithm" (TPA, see Boxes C (left) and D (right) in SI Figure 17). Those molecules may be impurities in the Carbamazepine standard, which nevertheless undergo similar photo-transformations.

Additional References

(1) Wilske, C.; Herzsprung, P.; Lechtenfeld, O. J.; Kamjunke, N.; von Tümpling, W. Photochemically Induced Changes of Dissolved Organic Matter in a Humic-Rich and Forested Stream. *Water* **2020**, *12* (2), 331. https://doi.org/10.3390/w12020331.

(2) Hu, A.; Li, L.; Huang, Y.; Fu, Q.-L.; Wang, D.; Zhang, W. Photochemical Transformation Mechanisms of Dissolved Organic Matters (DOM) Derived from Different Bio-Stabilization Sludge. *Environ. Int.* **2022**, *169*, 107534. https://doi.org/10.1016/j.envint.2022.107534.

(3) Zhang, B.; Wang, X.; Fang, Z.; Wang, S.; Shan, C.; Wei, S.; Pan, B. Unravelling Molecular Transformation of Dissolved Effluent Organic Matter in UV/H2O2, UV/Persulfate, and UV/Chlorine Processes Based on FT-ICR-MS Analysis. *Water Res.* **2021**, *199*, 117158. https://doi.org/10.1016/j.watres.2021.117158.

(4) Stedmon, C. A.; Markager, S.; Tranvik, L.; Kronberg, L.; Slätis, T.; Martinsen, W. Photochemical Production of Ammonium and Transformation of Dissolved Organic Matter in the Baltic Sea. *Mar. Chem.* **2007**, *104* (3), 227–240. https://doi.org/10.1016/j.marchem.2006.11.005.

(5) Zuo, Y.; Jones, R. D. Photochemistry of Natural Dissolved Organic Matter in Lake and Wetland Waters—Production of Carbon Monoxide. *Water Res.* **1997**, *31* (4), 850–858. https://doi.org/10.1016/S0043-1354(96)00316-8.

(6) Mesfioui, R.; Abdulla, H. A. N.; Hatcher, P. G. Photochemical Alterations of Natural and Anthropogenic Dissolved Organic Nitrogen in the York River. *Environ. Sci. Technol.* **2015**, *49* (1), 159–167. https://doi.org/10.1021/es504095c.

(7) Herzsprung, P.; Hertkorn, N.; Friese, K.; Schmitt-Kopplin, P. Photochemical Degradation of Natural Organic Sulfur Compounds (CHOS) from Iron-Rich Mine Pit Lake Pore Waters – an Initial Understanding from Evaluation of Single-Elemental Formulae Using Ultra-High-Resolution Mass Spectrometry. *Rapid Commun. Mass Spectrom.* **2010**, *24* (19), 2909–2924. https://doi.org/10.1002/rcm.4719.

(8) Zhang, B.; Shan, C.; Wang, S.; Fang, Z.; Pan, B. Unveiling the Transformation of Dissolved Organic Matter during Ozonation of Municipal Secondary Effluent Based on FT-ICR-MS and Spectral Analysis. *Water Res.* **2021**, *188*, 116484. https://doi.org/10.1016/j.watres.2020.116484.

(9) Raeke, J.; Lechtenfeld, O. J.; Seiwert, B.; Meier, T.; Riemenschneider, C.; Reemtsma, T. Photochemically Induced Bound Residue Formation of Carbamazepine with Dissolved Organic Matter. *Environ. Sci. Technol.* **2017**, *51* (10), 5523–5530. https://doi.org/10.1021/acs.est.7b00823.

(10) Privat, E.; Sowers, L. C. Photochemical Deamination and Demethylation of 5-Methylcytosine. *Chem. Res. Toxicol.* **1996**, *9* (4), 745–750. https://doi.org/10.1021/tx950182o.

(11) F. Nikitas, N.; Ioannis Tzaras, D.; Triandafillidi, I.; G. Kokotos, C. Photochemical Oxidation of Benzylic Primary and Secondary Alcohols Utilizing Air as the Oxidant. *Green Chem.* **2020**, *22* (2), 471–477. https://doi.org/10.1039/C9GC03000J.

(12) Hideko, K.; Hessler Bittl, D. P.; Fumihiro, M.; Yang, W.; Teruo, M. Photochemical Hydrogen Abstraction by Benzophenones from Hydrogen Donors in the Solid State. *J. Photochem. Photobiol. Chem.* **1995**, *86* (1), 171–176. https://doi.org/10.1016/1010-6030(94)03942-N.

(13) Hajimohammadi, M.; Schwarzinger, C.; Knör, G. Controlled Multistep Oxidation of Alcohols and Aldehydes to Carboxylic Acids Using Air, Sunlight and a Robust Metalloporphyrin Sensitizer with a PH-Switchable Photoreactivity. *RSC Adv.* **2012**, *2* (8), 3257–3260. https://doi.org/10.1039/C2RA01076C.
(14) Matykiewiczová, N.; Kurková, R.; Klánová, J.; Klán, P. Photochemically Induced Nitration and Hydroxylation of Organic Aromatic Compounds in the Presence of Nitrate or Nitrite in Ice. *J. Photochem. Photobiol. Chem.* **2007**, *187* (1), 24–32. https://doi.org/10.1016/j.jphotochem.2006.09.008.
(15) Hu, X.; Zhang, G.; Bu, F.; Lei, A. Visible-Light-Mediated Anti-Markovnikov Hydration of Olefins. *ACS Catal.* **2017**, *7* (2), 1432–1437. https://doi.org/10.1021/acscatal.6b03388.
(16) Forjan, M.; Zgrablić, G.; Vdović, S.; Šekutor, M.; Basarić, N.; Kabacinski, P.; Pashaki, M. N. H.; Frey, H.-M.; Cannizzo, A.; Cerullo, G. Photogeneration of Quinone Methide from Adamantylphenol in an Ultrafast Non-Adiabatic Dehydration Reaction. *Phys. Chem. Chem. Phys.* **2022**, *24* (7), 4384–4393. https://doi.org/10.1039/D1CP05690E.
(17) Ossola, R.; Gruseck, R.; Houska, J.; Manfrin, A.; Vallieres, M.; McNeill, K. Photochemical Production of Carbon Monoxide from Dissolved Organic Matter: Role of Lignin Methoxyarene Functional Groups. *Environ. Sci. Technol.* **2022**, *56* (18), 13449–13460. https://doi.org/10.1021/acs.est.2c03762.
(18) Xie, H.; Zafiriou, O. C.; Cai, W.-J.; Zepp, R. G.; Wang, Y. Photooxidation and Its Effects on the Carboxyl Content of Dissolved Organic Matter in Two Coastal Rivers in the Southeastern United States. *Environ. Sci. Technol.* **2004**, *38* (15), 4113–4119. https://doi.org/10.1021/es035407t.
(19) Klán, P.; Růžička, R.; Heger, D.; Literák, J.; Kulhánek, P.; Loupy, A. Temperature-Sensitive Photochemical Aromatic Substitution on 4-Nitroanisole. *Photochem. Photobiol. Sci.* **2002**, *1* (12), 1012–1016. https://doi.org/10.1039/b209010d.
(20) Elgouch, J.; Catastini, C.; Lavedrine, B.; Guyot, G.; Sarakha, M. Photolysis of Aqueous Solutions of Aniline-2,5-Disulfonic Acid: Steady State and Laser Flash Photolysis Studies. *Photochem. Photobiol. Sci.* **2002**, *1* (7), 514–519. https://doi.org/10.1039/b202582e.
(21) Ossola, R.; Clerc, B.; McNeill, K. Mechanistic Insights into Dissolved Organic Sulfur Photomineralization through the Study of Cysteine Sulfinic Acid. *Environ. Sci. Technol.* **2020**, *54* (20), 13066–13076. https://doi.org/10.1021/acs.est.0c04340.
